# Supplementary material for: Mapping health service coverage inequalities in Africa: a scoping review protocol
Source: BMJ Open. 2023 May 30;13(5):e068903. doi: 10.1136/bmjopen-2022-068903 (PMC10255155; doi:10.1136/bmjopen-2022-068903)
Supplement: Supplementary data [file bmjopen-2022-068903supp002.pdf]

Appendix 2: Health Service Coverage Indicators

**Indicators for measuring progress towards reproductive, maternal, newborn and child health (RMNCH)**
**Family planning** (e.g., demand satisfied with modern contraception methods in women)
**Antenatal care** (e.g., women who received antenatal care four or more time)
**Delivery care** (e.g., births attended by skilled health personnel)
**Postnatal care** (e.g., women or newborn that received postnatal care services after birth)
**Child immunization** (e.g., children aged 1 year who have received three doses of diphtheria, tetanus, and pertussis vaccine (DPT3) vaccine)
**Health-seeking behaviour for pneumonia** (e.g., children with suspected pneumonia who have sought care)
**Indicators for measuring progress towards infectious diseases**
**Tuberculosis treatment** (e.g., people who received effective tuberculosis treatment)
**Human immunodeficiency virus (HIV) therapy** (e.g., people with HIV receiving antiretroviral treatment)
**Use of insecticide-treated bed nets for malaria prevention** (e.g., people sleeping under insecticide-treated nets)
**Adequate sanitation** (e.g., households using at least basic sanitation facilities)
**Neglected tropical diseases treatment and care** (e.g., people required treatment for any of the neglected tropical diseases). Neglected tropical diseases are a diverse group of 20 conditions including: Buruli ulcer, Chagas disease, dengue and chikungunya, dracunculiasis (Guinea-worm disease), echinococcosis, foodborne trematodiases, human African trypanosomiasis (sleeping sickness), leishmaniasis, leprosy (Hansen's disease), lymphatic filariasis, mycetoma, chromoblastomycosis and other deep mycoses, onchocerciasis (river blindness), rabies, scabies and other ectoparasitoses, schistosomiasis, soil-transmitted helminthiases, snakebite envenoming, taeniasis/cysticercosis, trachoma, and yaws and other endemic treponematoses.
**Indicators for measuring progress towards non-communicable diseases**
**Prevention and treatment of raised blood pressure** (e.g., prevalence of raised blood pressure regardless of treatment status)
**Prevention and treatment of raised blood glucose** (e.g., mean fasting plasma glucose)
**Cervical cancer screening** (e.g., prevalence of cervical cancer screening at aged 30-49)
**Tobacco (non-)smoking** (e.g., prevalence of tobacco use among adults aged at least 15 years)

Karamagi HC, *et al. BMJ Open* 2023; 13:e068903. doi: 10.1136/bmjopen-2022-068903
