# Supplementary material for: Mapping health service coverage inequalities in Africa: a scoping review protocol
Source: BMJ Open. 2023 May 30;13(5):e068903. doi: 10.1136/bmjopen-2022-068903 (PMC10255155; doi:10.1136/bmjopen-2022-068903)
Supplement: Supplementary data [file bmjopen-2022-068903supp003.pdf]

Appendix 3. Electronic Search Strategies for Databases

MEDLINE via Ovid (searched on August 29, 2022; Period: 1946 - August 26, 2022)

Concept		Keyword	umber	Result
**Inequality**	**Controlled Vocabulary**	exp "gender identity"/ or exp "sexual and gender minorities"/ or exp "disabled persons"/ or exp geriatrics/ or "health equity"/ or "health inequities"/ or "healthcare disparities"/ or "health status disparities"/ or "health services accessibility"/ or "sex factors"/ or women/ or men/ or "residence characteristics"/ or "urban population"/ or "rural population"/ or "cultural characteristics"/ or "cultural diversity"/ or religion/ or "social discrimination"/ or "socioeconomic factors"/ or "vulnerable populations"/ or "social class"/ or "minority groups"/ or "ethnic and racial minorities"/ or "educational status"/ or unemployment/	1	884,458
	**Free Text**	(equit* or inequit* or inequalit* or disparit* or equalit* or deprivation or gini or "concentration index" or ((health* or medical) adj3 (availabl* or access*)) or (gender adj (difference? or identit* or role?)) or (sex adj (disparit* or difference? or role?)) or ((wom#n* or m#n*) adj role?) or "sexual minorit*" or "gender minorit*" or LGBT* or 2SLGBT* or urban or rural or ethnic* or religi* or ((social* or socio-economic or socioeconomic or economic or structural or material) adj3 (advantage* or disadvantage* or exclude* or exclusion or include* or inclusion or status or position or gradient* or hierarch* or class* or determinant*)) or (health adj3 (gap* or gradient* or hierarch*)) or SES or SEP or sociodemographic* or socio-demographic* or income or wealth* or poverty or "educational status" or "educational level?" or "level? of education" or "educational attainment" or ((well or better or higher or worse or less) adj (educated)) or unemploy* or "home owner*" or tenure or affluen* or "well off" or "better off" or "worse off").ti,ab,kw,kf	2	1,197,912
	**All**	1 or 2	3	1,739,958
**Health Service Coverage**	**Controlled Vocabulary**	"universal health care"/	4	243
	**Free Text**	(UHC or (universal adj2 (coverage or health* or care or access))).ti,ab,kw,kf	5	12,120
	**All**	4 or 5	6	12,186
**Africa**	**Controlled Vocabulary**	exp africa/ not ("guinea pig" or "guinea pigs" or "aspergillus niger").ti,ab,kw,kf,cp,in	7	308,111
	**Free Text**	((africa* or algeria or algerie or angola or benin or dahomey or botswana or bechuanaland or "burkina faso" or "burkina fasso" or "upper volta" or "haute volta" or burundi or urundi or cameroon or cameroun or "canary islands" or "cape verde" or cabo verde or "ubangi shari" or chad or tchad or comoros or "comoro islands" or comores or mayotte or congo or zaire or djibouti or "french somaliland" or egypt or "united arab republic" or eritrea or eswatini or swaziland or ethiopia or gabon or "gabonese republic" or gambia or ghana or "gold coast" or guinea or "ivory coast" or "cote d?ivoire" or "cote d' ivoire" or kenya or lesotho or basutoland or liberia or libya or libia or jamahiriya or jamahiryia or madagascar or "malagasy republic" or malawi or nyasaland or mali or mauritania or mauritius or morocco or ifni or mozambique or "portuguese east africa" or mocambique or namibia or niger or nigeria or principe or "sao tome" or reunion or rwanda or ruanda or senegal or seychelles or "sierra leone" or somalia or "south africa" or "st helena" or sudan or tanzania or tanganyika or togo or "togolese republic" or tunisia or tunisie or uganda or "western sahara" or zambia or zimbabwe or "indian ocean islands" or sahara or magreb or magrib) not ("guinea pig" or "guinea pigs" or "aspergillus niger")).ti,ab,kw,kf,cp,in	8	885,575
	**All**	7 or 8	9	921,268
**Total result**		**3 AND 6 AND 9**	**10**	**1,924**

Karamagi HC, *et al. BMJ Open* 2023; 13:e068903. doi: 10.1136/bmjopen-2022-068903

Embase via Elsevier (searched on August 29, 2022)

Concept		Keyword	umber	Result
**Inequality**	**Controlled Vocabulary**	'gender identity'/exp or 'sexual and gender minority'/exp or 'disabled person'/exp or 'disabled person'/exp or 'health equity'/de or 'health disparity'/de or 'health care access'/de or 'health care availability'/de or 'sex difference'/de or 'female'/de or 'male'/de or 'residence characteristics'/de or 'urban population'/de or 'rural population'/de or 'cultural factor'/de or 'cultural diversity'/de or 'religion'/de or 'social discrimination'/de or 'socioeconomics'/de or 'vulnerable population'/de or 'disadvantaged population'/de or 'social class'/de or 'minority group'/de or 'ethnic group'/de or 'educational status'/de or 'unemployment'/de	#1	15,833,425
	**Free Text**	(equit* or inequit* or inequalit* or disparit* or equalit* or deprivation or gini or "concentration index" or ((health* or medical) NEAR/3 (availabl* or access*)) or (gender NEXT/1 (difference$ or identit* or role$)) or (sex NEXT/1 (disparit* or difference$ or role$)) or ((wom?n* or m?n*) NEXT/1 role$) or "sexual minorit*" or "gender minorit*" or LGBT* or 2SLGBT* or urban or rural or ethnic* or religi* or ((social* or socio-economic or socioeconomic or economic or structural or material) NEAR/3 (advantage* or disadvantage* or exclude* or exclusion or include* or inclusion or status or position or gradient* or hierarch* or class* or determinant*)) or (health NEAR/3 (gap* or gradient* or hierarch*)) or SES or SEP or sociodemographic* or socio-demographic* or income or wealth* or poverty or "educational status" or "educational level$" or "level$ of education" or "educational attainment" or ((well or better or higher or worse or less) NEXT/1 (educated)) or unemploy* or "home owner*" or tenure or affluen* or "well off" or "better off" or "worse off"):ti,ab,kw	#2	1,527,2697
	**All**	1 or 2	#3	16,237,327
**Health Service Coverage**	**Controlled Vocabulary**	'universal health care'/de	#4	600
	**Free Text**	(UHC or (universal NEAR/2 (coverage or health* or care or access))):ti,ab,kw	#5	14,728
	**All**	4 or 5	#6	14,913
**Africa**	**Controlled Vocabulary**	'africa'/exp not ("guinea pig" or "guinea pigs" or "aspergillus niger"):ti,ab,kw,ca,ad,ff	#7	397,519
	**Free Text**	((africa* or algeria or algerie or angola or benin or dahomey or botswana or bechuanaland or "burkina faso" or "burkina fasso" or "upper volta" or "haute volta" or burundi or urundi or cameroon or cameroun or "canary islands" or "cape verde" or cabo verde or "ubangi shari" or chad or tchad or comoros or "comoro islands" or comores or mayotte or congo or zaire or djibouti or "french somaliland" or egypt or "united arab republic" or eritrea or eswatini or swaziland or ethiopia or gabon or "gabonese republic" or gambia or ghana or "gold coast" or guinea or "ivory coast" or "cote d$ivoire" or "cote d? ivoire" or kenya or lesotho or basutoland or liberia or libya or libia or jamahiriya or jamahiryia or madagascar or "malagasy republic" or malawi or nyasaland or mali or mauritania or mauritius or morocco or ifni or mozambique or "portuguese east africa" or mocambique or namibia or niger or nigeria or principe or "sao tome" or reunion or rwanda or ruanda or senegal or seychelles or "sierra leone" or somalia or "south africa" or "st helena" or sudan or tanzania or tanganyika or togo or "togolese republic" or tunisia or tunisie or uganda or "western sahara" or zambia or zimbabwe or "indian ocean islands" or sahara or magreb or magrib) not ("guinea pig" or "guinea pigs" or "aspergillus niger")):ti,ab,kw,ca,ad,ff	#8	859,620
	**All**	7 or 8	#9	960,563
**Total result**		**#3 AND #6 AND #9**	**#10**	2,586

Web of Science (searched on August 29, 2022)

Concept		Keyword	umber	Result
**Inequality**	**Free Text**	TS=(equit* or inequit* or inequalit* or disparit* or equalit* or deprivation or gini or "concentration index" or ((health* or medical) NEAR/2 (availabl* or access*)) or (gender NEAR/0 (difference$ or identit* or role$)) or (sex NEAR/0 (disparit* or difference$ or role$)) or ((wom?n* or man* or men*) NEAR/0 role$) or "sexual minorit*" or "gender minorit*" or LGBT* or 2SLGBT* or urban or rural or ethnic* or religi* or ((social* or socio-economic or socioeconomic or economic or structural or material) NEAR/2 (advantage* or disadvantage* or exclude* or exclusion or include* or inclusion or status or position or gradient* or hierarch* or class* or determinant*)) or (health NEAR/2 (gap* or gradient* or hierarch*)) or SES or SEP or sociodemographic* or socio-demographic* or income or wealth* or poverty or "educational status" or "educational level$" or "level$ of education" or "educational attainment" or ((well or better or higher or worse or less) NEAR/0 (educated)) or unemploy* or "home owner*" or tenure or affluen* or "well off" or "better off" or "worse off")	#1	3,056,690
**Health Service Coverage**	**Free Text**	TS=(UHC or (universal NEAR/1 (coverage or health* or care or access)))	#2	15,460
**Africa**	**Free Text**	TS=((africa* or algeria or algerie or angola or benin or dahomey or botswana or bechuanaland or "burkina faso" or "burkina fasso" or "upper volta" or "haute volta" or burundi or urundi or cameroon or cameroun or "canary islands" or "cape verde" or cabo verde or "ubangi shari" or chad or tchad or comoros or "comoro islands" or comores or mayotte or congo or zaire or djibouti or "french somaliland" or egypt or "united arab republic" or eritrea or eswatini or swaziland or ethiopia or gabon or "gabonese republic" or gambia or ghana or "gold coast" or guinea or "ivory coast" or "cote d$ivoire" or "cote d' ivoire" or kenya or lesotho or basutoland or liberia or libya or libia or jamahiriya or jamahiryia or madagascar or "malagasy republic" or malawi or nyasaland or mali or mauritania or mauritius or morocco or ifni or mozambique or "portuguese east africa" or mocambique or namibia or niger or nigeria or principe or "sao tome" or reunion or rwanda or ruanda or senegal or seychelles or "sierra leone" or somalia or "south africa" or "st helena" or sudan or tanzania or tanganyika or togo or "togolese republic" or tunisia or tunisie or uganda or "western sahara" or zambia or zimbabwe or "indian ocean islands" or sahara or magreb or magrib) not ("guinea pig" or "guinea pigs" or "aspergillus niger")) or<br>CU=((africa* or algeria or algerie or angola or benin or dahomey or botswana or bechuanaland or "burkina faso" or "burkina fasso" or "upper volta" or "haute volta" or burundi or urundi or cameroon or cameroun or "canary islands" or "cape verde" or cabo verde or "ubangi shari" or chad or tchad or comoros or "comoro islands" or comores or mayotte or congo or zaire or djibouti or "french somaliland" or egypt or "united arab republic" or eritrea or eswatini or swaziland or ethiopia or gabon or "gabonese republic" or gambia or ghana or "gold coast" or guinea or "ivory coast" or "cote d$ivoire" or "cote d' ivoire" or kenya or lesotho or basutoland or liberia or libya or libia or jamahiriya or jamahiryia or madagascar or "malagasy republic" or malawi or nyasaland or mali or mauritania or mauritius or morocco or ifni or mozambique or "portuguese east africa" or mocambique or namibia or niger or nigeria or principe or "sao tome" or reunion or rwanda or ruanda or senegal or seychelles or "sierra leone" or somalia or "south africa" or "st helena" or sudan or tanzania or tanganyika or togo or "togolese republic" or tunisia or tunisie or uganda or "western sahara" or zambia or zimbabwe or "indian ocean islands" or sahara or magreb or magrib) not ("guinea pig" or "guinea pigs" or "aspergillus niger")) or<br>OG=((africa* or algeria or algerie or angola or benin or dahomey or botswana or bechuanaland or "burkina faso" or "burkina fasso" or "upper volta" or "haute volta" or burundi or urundi or cameroon or cameroun or "canary islands" or "cape verde" or cabo verde or "ubangi shari" or chad or tchad or comoros or "comoro islands" or comores or mayotte or congo or zaire or djibouti or "french somaliland" or egypt or "united arab republic" or eritrea or eswatini or swaziland or ethiopia or gabon or "gabonese republic" or gambia or ghana or "gold coast" or guinea or "ivory coast" or "cote d$ivoire" or "cote d' ivoire" or kenya or lesotho or basutoland or liberia or libya or libia or jamahiriya or jamahiryia or madagascar or "malagasy republic" or malawi	#3	2,204,863

		or nyasaland or mali or mauritania or mauritius or morocco or ifni or mozambique or "portuguese east africa" or mocambique or namibia or niger or nigeria or principe or "sao tome" or reunion or rwanda or ruanda or senegal or seychelles or "sierra leone" or somalia or "south africa" or "st helena" or sudan or tanzania or tanganyika or togo or "togolese republic" or tunisia or tunisie or uganda or "western sahara" or zambia or zimbabwe or "indian ocean islands" or sahara or magreb or magrib) not ("guinea pig" or "guinea pigs" or "aspergillus niger"))		
**Total result**		**#1 AND #2 AND #3**	**#4**	**2,145**

CINAHL via EBSCOhost (searched on August 19, 2022)

Concept		Keyword	umber	Result
**Inequality**	**Controlled Vocabulary**	(MH "gender identity+") or (MH "sexual and gender minorities+") or (MH "persons with disabilities+") or (MH "geriatrics+") or (MH "health inequities") or (MH "healthcare disparities") or (MH "health status disparities") or (MH "health services accessibility") or (MH "sex factors") or (MH "women") or (MH "men") or (MH "residence characteristics") or (MH "urban population") or (MH "rural population") or (MH "cultural diversity") or (MH "religion and religions") or (MH "discrimination") or (MH "socioeconomic factors") or (MH "special populations") or (MH "social class") or (MH "minority groups") or (MH "ethnic groups") or (MH "educational status") or (MH "unemployment")	S1	544,616
	**Free Text**	TI (equit* or inequit* or inequalit* or disparit* or equalit* or deprivation or gini or "concentration index" or ((health* or medical) N2 (availabl* or access*)) or (gender W1 (difference# or identit* or role#)) or (sex W1 (disparit* or difference# or role#)) or ((wom?n* or m?n*) W1 role#) or "sexual minorit*" or "gender minorit*" or LGBT* or 2SLGBT* or urban or rural or ethnic* or religi* or ((social* or socio-economic or socioeconomic or economic or structural or material) N2 (advantage* or disadvantage* or exclude* or exclusion or include* or inclusion or status or position or gradient* or hierarch* or class* or determinant*)) or (health N2 (gap* or gradient* or hierarch*)) or SES or SEP or sociodemographic* or socio-demographic* or income or wealth* or poverty or "educational status" or "educational level#" or "level# of education" or "educational attainment" or ((well or better or higher or worse or less) W1 (educated)) or unemploy* or "home owner*" or tenure or affluen* or "well off" or "better off" or "worse off") OR<br>AB (equit* or inequit* or inequalit* or disparit* or equalit* or deprivation or gini or "concentration index" or ((health* or medical) N2 (availabl* or access*)) or (gender W1 (difference# or identit* or role#)) or (sex W1 (disparit* or difference# or role#)) or ((wom?n* or m?n*) W1 role#) or "sexual minorit*" or "gender minorit*" or LGBT* or 2SLGBT* or urban or rural or ethnic* or religi* or ((social* or socio-economic or socioeconomic or economic or structural or material) N2 (advantage* or disadvantage* or exclude* or exclusion or include* or inclusion or status or position or gradient* or hierarch* or class* or determinant*)) or (health N2 (gap* or gradient* or hierarch*)) or SES or SEP or sociodemographic* or socio-demographic* or income or wealth* or poverty or "educational status" or "educational level#" or "level# of education" or "educational attainment" or ((well or better or higher or worse or less) W1 (educated)) or unemploy* or "home owner*" or tenure or affluen* or "well off" or "better off" or "worse off") OR<br>SU (equit* or inequit* or inequalit* or disparit* or equalit* or deprivation or gini or "concentration index" or ((health* or medical) N2 (availabl* or access*)) or (gender W1 (difference# or identit* or role#)) or (sex W1 (disparit* or difference# or role#)) or ((wom?n* or m?n*) W1 role#) or "sexual minorit*" or "gender minorit*" or LGBT* or 2SLGBT* or urban or rural or ethnic* or religi* or ((social* or socio-economic or socioeconomic or economic or structural or material) N2 (advantage* or disadvantage* or exclude* or exclusion or include* or inclusion or status or position or gradient* or hierarch* or class* or determinant*)) or (health N2 (gap* or gradient* or hierarch*)) or SES or SEP or sociodemographic* or socio-demographic* or income or wealth* or poverty or "educational status" or "educational level#" or "level# of education" or "educational attainment" or ((well or better or higher or worse or less) W1 (educated)) or unemploy* or "home owner*" or tenure or affluen* or "well off" or "better off" or "worse off")	S2	654,635
	**All**	S1 or S2	S3	874,600
**Health Service Coverage**	**Controlled Vocabulary**	MH "universal health care"	S4	552
	**Free Text**	TI (UHC or (universal N1 (coverage or health* or care or access))) OR AB (UHC or (universal N1 (coverage or health* or care or access))) OR SU (UHC or (universal N1 (coverage or health* or care or access)))	S5	6,489
	**All**	S4 or S5	S6	6,489

Page **5** of **9**

**Africa**	**Controlled Vocabulary**	(MH "africa+") not (TI ("guinea pig" or "guinea pigs" or "aspergillus niger") OR AB ("guinea pig" or "guinea pigs" or "aspergillus niger") OR SU ("guinea pig" or "guinea pigs" or "aspergillus niger") OR AF ("guinea pig" or "guinea pigs" or "aspergillus niger"))	S7	96,591
	**Free Text**	TI ((africa* or algeria or algerie or angola or benin or dahomey or botswana or bechuanaland or "burkina faso" or "burkina fasso" or "upper volta" or "haute volta" or burundi or urundi or cameroon or cameroun or "canary islands" or "cape verde" or cabo verde or "ubangi shari" or chad or tchad or comoros or "comoro islands" or comores or mayotte or congo or zaire or djibouti or "french somaliland" or egypt or "united arab republic" or eritrea or eswatini or swaziland or ethiopia or gabon or "gabonese republic" or gambia or ghana or "gold coast" or guinea or "ivory coast" or "cote d#ivoire" or "cote d' ivoire" or kenya or lesotho or basutoland or liberia or libya or libia or jamahiriya or jamahiryia or madagascar or "malagasy republic" or malawi or nyasaland or mali or mauritania or mauritius or morocco or ifni or mozambique or "portuguese east africa" or mocambique or namibia or niger or nigeria or principe or "sao tome" or reunion or rwanda or ruanda or senegal or seychelles or "sierra leone" or somalia or "south africa" or "st helena" or sudan or tanzania or tanganyika or togo or "togolese republic" or tunisia or tunisie or uganda or "western sahara" or zambia or zimbabwe or "indian ocean islands" or sahara or magreb or magrib) not ("guinea pig" or "guinea pigs" or "aspergillus niger")) OR<br>AB ((africa* or algeria or algerie or angola or benin or dahomey or botswana or bechuanaland or "burkina faso" or "burkina fasso" or "upper volta" or "haute volta" or burundi or urundi or cameroon or cameroun or "canary islands" or "cape verde" or cabo verde or "ubangi shari" or chad or tchad or comoros or "comoro islands" or comores or mayotte or congo or zaire or djibouti or "french somaliland" or egypt or "united arab republic" or eritrea or eswatini or swaziland or ethiopia or gabon or "gabonese republic" or gambia or ghana or "gold coast" or guinea or "ivory coast" or "cote d#ivoire" or "cote d' ivoire" or kenya or lesotho or basutoland or liberia or libya or libia or jamahiriya or jamahiryia or madagascar or "malagasy republic" or malawi or nyasaland or mali or mauritania or mauritius or morocco or ifni or mozambique or "portuguese east africa" or mocambique or namibia or niger or nigeria or principe or "sao tome" or reunion or rwanda or ruanda or senegal or seychelles or "sierra leone" or somalia or "south africa" or "st helena" or sudan or tanzania or tanganyika or togo or "togolese republic" or tunisia or tunisie or uganda or "western sahara" or zambia or zimbabwe or "indian ocean islands" or sahara or magreb or magrib) not ("guinea pig" or "guinea pigs" or "aspergillus niger")) OR<br>SU ((africa* or algeria or algerie or angola or benin or dahomey or botswana or bechuanaland or "burkina faso" or "burkina fasso" or "upper volta" or "haute volta" or burundi or urundi or cameroon or cameroun or "canary islands" or "cape verde" or cabo verde or "ubangi shari" or chad or tchad or comoros or "comoro islands" or comores or mayotte or congo or zaire or djibouti or "french somaliland" or egypt or "united arab republic" or eritrea or eswatini or swaziland or ethiopia or gabon or "gabonese republic" or gambia or ghana or "gold coast" or guinea or "ivory coast" or "cote d#ivoire" or "cote d' ivoire" or kenya or lesotho or basutoland or liberia or libya or libia or jamahiriya or jamahiryia or madagascar or "malagasy republic" or malawi or nyasaland or mali or mauritania or mauritius or morocco or ifni or mozambique or "portuguese east africa" or mocambique or namibia or niger or nigeria or principe or "sao tome" or reunion or rwanda or ruanda or senegal or seychelles or "sierra leone" or somalia or "south africa" or "st helena" or sudan or tanzania or tanganyika or togo or "togolese republic" or tunisia or tunisie or uganda or "western sahara" or zambia or zimbabwe or "indian ocean islands" or sahara or magreb or magrib) not ("guinea pig" or "guinea pigs" or "aspergillus niger")) OR<br>AF ((africa* or algeria or algerie or angola or benin or dahomey or botswana or bechuanaland or "burkina faso" or "burkina fasso" or "upper volta" or "haute volta" or burundi or urundi or cameroon or cameroun or "canary islands" or "cape verde" or cabo verde or "ubangi shari" or chad or tchad or comoros or "comoro islands" or comores or mayotte or congo or zaire or djibouti or "french somaliland" or egypt or "united arab republic" or eritrea or eswatini or swaziland or ethiopia or gabon or "gabonese republic" or gambia or ghana or "gold coast" or guinea or "ivory coast" or "cote d#ivoire" or "cote d' ivoire" or kenya or lesotho or basutoland or liberia or libya or libia or jamahiriya or jamahiryia or madagascar or "malagasy republic" or malawi or nyasaland or mali or mauritania or mauritius or morocco or ifni or mozambique or "portuguese east africa" or mocambique or	S8	210,457

		namibia or niger or nigeria or principe or "sao tome" or reunion or rwanda or ruanda or senegal or seychelles or "sierra leone" or somalia or "south africa" or "st helena" or sudan or tanzania or tanganyika or togo or "togolese republic" or tunisia or tunisie or uganda or "western sahara" or zambia or zimbabwe or "indian ocean islands" or sahara or magreb or magrib) not ("guinea pig" or "guinea pigs" or "aspergillus niger"))		
	**All**	S7 or S8	S9	210,575
**Total result**		**S3 AND S6 AND S9**	**S10**	**899**

PsycINFO via Ovid (searched on August 29, 2022; Period: 1806 - August Week 3, 2022)

Concept		Keyword	umber	Result
**Inequality**	**Controlled Vocabulary**	exp "gender identity"/ or exp "sexual minority groups"/ or exp disabilities/ or exp geriatrics/ or equity/ or "healthcare disparities"/ or "health care access"/ or "human sex differences"/ or "human females"/ or "human males"/ or neighborhoods/ or ghettoes/ or poverty areas/ or "rural environments"/ or "urban environments"/ or "minority groups"/ or "racial and ethnic differences"/ or "sociocultural factors"/ or "cross cultural differences"/ or "cultural identity"/ or "ethnic identity"/ or religion/ or "religious groups"/ or "social discrimination"/ or "socioeconomic factors"/ or "socioeconomic status"/ or "economic inequality"/ or "susceptibility (disorders)"/ or "at risk populations"/ or "social class"/ or disadvantaged/ or "educational attainment level"/ or unemployment/	1	619,023
	**Free Text**	(equit* or inequit* or inequalit* or disparit* or equalit* or deprivation or gini or "concentration index" or ((health* or medical) adj3 (availabl* or access*)) or (gender adj (difference? or identit* or role?)) or (sex adj (disparit* or difference? or role?)) or ((wom#n* or m#n*) adj role?) or "sexual minorit*" or "gender minorit*" or LGBT* or 2SLGBT* or urban or rural or ethnic* or religi* or ((social* or socio-economic or socioeconomic or economic or structural or material) adj3 (advantage* or disadvantage* or exclude* or exclusion or include* or inclusion or status or position or gradient* or hierarch* or class* or determinant*)) or (health adj3 (gap* or gradient* or hierarch*)) or SES or SEP or sociodemographic* or socio-demographic* or income or wealth* or poverty or "educational status" or "educational level?" or "level? of education" or "educational attainment" or ((well or better or higher or worse or less) adj (educated)) or unemploy* or "home owner*" or tenure or affluen* or "well off" or "better off" or "worse off").ti,ab,id.	2	698,649
	**All**	1 or 2	3	1,036,490
**Health Service Coverage**	**Free Text**	(UHC or (universal adj2 (coverage or health* or care or access))).ti,ab,id.	4	1,840
**Africa**	**Free Text**	((africa* or algeria or algerie or angola or benin or dahomey or botswana or bechuanaland or "burkina faso" or "burkina fasso" or "upper volta" or "haute volta" or burundi or urundi or cameroon or cameroun or "canary islands" or "cape verde" or cabo verde or "ubangi shari" or chad or tchad or comoros or "comoro islands" or comores or mayotte or congo or zaire or djibouti or "french somaliland" or egypt or "united arab republic" or eritrea or eswatini or swaziland or ethiopia or gabon or "gabonese republic" or gambia or ghana or "gold coast" or guinea or "ivory coast" or "cote d?ivoire" or "cote d' ivoire" or kenya or lesotho or basutoland or liberia or libya or libia or jamahiriya or jamahiryia or madagascar or "malagasy republic" or malawi or nyasaland or mali or mauritania or mauritius or morocco or ifni or mozambique or "portuguese east africa" or mocambique or namibia or niger or nigeria or principe or "sao tome" or reunion or rwanda or ruanda or senegal or seychelles or "sierra leone" or somalia or "south africa" or "st helena" or sudan or tanzania or tanganyika or togo or "togolese republic" or tunisia or tunisie or uganda or "western sahara" or zambia or zimbabwe or "indian ocean islands" or sahara or magreb or magrib) not ("guinea pig" or "guinea pigs" or "aspergillus niger")).ti,ab,id,in	5	141,449
**Total result**		**3 AND 4 AND 5**	**6**	**168**

Cochrane Library (searched on August 29, 2022)

Concept		Keyword	umber	Result
**Inequality**	**Controlled Vocabulary**	[mh "gender identity"] or [mh "sexual and gender minorities"] or [mh "disabled persons"] or [mh "geriatrics"] or [mh ^"health equity"] or [mh ^"health inequities"] or [mh ^"healthcare disparities"] or [mh ^"health status disparities"] or [mh ^"health services accessibility"] or [mh ^"sex factors"] or [mh ^"women"] or [mh ^"men"] or [mh ^"residence characteristics"] or [mh ^"urban population"] or [mh ^"rural population"] or [mh ^"cultural characteristics"] or [mh ^"cultural diversity"] or [mh ^"religion"] or [mh ^"social discrimination"] or [mh ^"socioeconomic factors"] or [mh ^"vulnerable populations"] or [mh ^"social class"] or [mh ^"minority groups"] or [mh ^"ethnic and racial minorities"] or [mh ^"educational status"] or [mh ^"unemployment"]	#1	18,168
	**Free Text**	(equit* or inequit* or inequalit* or disparit* or equalit* or deprivation or gini or "concentration index" or ((health* or medical) NEAR/2 (availabl* or access*)) or (gender NEXT (difference? or identit* or role?)) or (sex NEXT (disparit* or difference? or role?)) or ((wom?n* or m?n*) NEXT role?) or "sexual minorit*" or "gender minorit*" or LGBT* or 2SLGBT* or urban or rural or ethnic* or religi* or ((social* or socio-economic or socioeconomic or economic or structural or material) NEAR/2 (advantage* or disadvantage* or exclude* or exclusion or include* or inclusion or status or position or gradient* or hierarch* or class* or determinant*)) or (health NEAR/2 (gap* or gradient* or hierarch*)) or SES or SEP or sociodemographic* or socio-demographic* or income or wealth* or poverty or "educational status" or "educational level?" or "level? of education" or "educational attainment" or ((well or better or higher or worse or less) NEXT (educated)) or unemploy* or "home owner*" or tenure or affluen* or "well off" or "better off" or "worse off"):ti,ab,kw	#2	79,541
	**All**	#1 or #2	#3	87,932
**Health Service Coverage**	**Controlled Vocabulary**	[mh ^"universal health care"]	#4	0
	**Free Text**	(UHC or (universal NEAR/1 (coverage or health* or care or access))):ti,ab,kw	#5	306
	**All**	#4 or #5	#6	306
**Africa**	**Controlled Vocabulary**	[mh "africa"] not ("guinea pig" or "guinea pigs" or "aspergillus niger"):ti,ab,kw	#7	8,273
	**Free Text**	((africa* or algeria or algerie or angola or benin or dahomey or botswana or bechuanaland or "burkina faso" or "burkina fasso" or "upper volta" or "haute volta" or burundi or urundi or cameroon or cameroun or "canary islands" or "cape verde" or cabo verde or "ubangi shari" or chad or tchad or comoros or "comoro islands" or comores or mayotte or congo or zaire or djibouti or "french somaliland" or egypt or "united arab republic" or eritrea or eswatini or swaziland or ethiopia or gabon or "gabonese republic" or gambia or ghana or "gold coast" or guinea or "ivory coast" or "cote d?ivoire" or "cote d' ivoire" or kenya or lesotho or basutoland or liberia or libya or libia or jamahiriya or jamahiryia or madagascar or "malagasy republic" or malawi or nyasaland or mali or mauritania or mauritius or morocco or ifni or mozambique or "portuguese east africa" or mocambique or namibia or niger or nigeria or principe or "sao tome" or reunion or rwanda or ruanda or senegal or seychelles or "sierra leone" or somalia or "south africa" or "st helena" or sudan or tanzania or tanganyika or togo or "togolese republic" or tunisia or tunisie or uganda or "western sahara" or zambia or zimbabwe or "indian ocean islands" or sahara or magreb or magrib) not ("guinea pig" or "guinea pigs" or "aspergillus niger")):ti,ab,kw	#8	34,555
	**All**	#7 or #8	#9	34,555
**Total result**		**#3 AND #6 AND #9**	**#10**	**56**

Karamagi HC, *et al. BMJ Open* 2023; 13:e068903. doi: 10.1136/bmjopen-2022-068903
