# Supplementary material for: Mapping health service coverage inequalities in Africa: a scoping review protocol
Source: BMJ Open. 2023 May 30;13(5):e068903. doi: 10.1136/bmjopen-2022-068903 (PMC10255155; doi:10.1136/bmjopen-2022-068903)
Supplement: Author's manuscript [file bmjopen-2022-068903.draft_revisions.pdf]

BMJ Open is committed to open peer review. As part of this commitment we make the peer review history of every article we publish publicly available.

When an article is published we post the peer reviewers' comments and the authors' responses online. We also post the versions of the paper that were used during peer review. These are the versions that the peer review comments apply to.

The versions of the paper that follow are the versions that were submitted during the peer review process. They are not the versions of record or the final published versions. They should not be cited or distributed as the published version of this manuscript.

BMJ Open is an open access journal and the full, final, typeset and author-corrected version of record of the manuscript is available on our site with no access controls, subscription charges or pay-per-view fees (http://bmjopen.bmj.com).

If you have any questions on BMJ Open's open peer review process please email info.bmjopen@bmj.com

BMJ Open

Mapping the progress of achieving equity in health service coverage in Africa: a scoping review protocol

Journal:	*BMJ Open*
Manuscript ID	bmjopen-2022-068903
Article Type:	Protocol
Date Submitted by the Author:	06-Oct-2022
Complete List of Authors:	Karamagi, Humphrey Cyprian ; World Health Organization Regional Office for Africa,<br>Ben Charif, Ali; Université Laval,<br>Afriyie, Doris; Swiss Tropical and Public Health Institute, Epidemiology and Public Health; University of Basel,<br>SY, Sokona; World Health Organization Regional Office for Africa, Data, Analytics and Knowledge Management<br>kipruto, Hillary; World Health Organization Regional Office for Africa, Inter-Country Support Team for Eastern & Southern Africa<br>Oyelade, Taiwo; World Health Organization Regional Office for Africa<br>Droti, Benson; World Health Organization Regional Office for Africa
Keywords:	Protocols & guidelines < HEALTH SERVICES ADMINISTRATION & MANAGEMENT, PUBLIC HEALTH, STATISTICS & RESEARCH METHODS

SCHOLARONE™ Manuscripts

Mapping the progress of achieving equity in health service coverage in Africa: a scoping review protocol

Humphrey Cyprian KARAMAGI[1]*, Ali BEN CHARIF[2], Doris OSEI AFRIYIE[3], Sokona SY[1], Hillary Kipchumba KIPRUTO[4], Oyelade TAIWO[5], Benson DROTI[4]

Institutional addresses

[1]Data Analytics and Knowledge Management, World Health Organization (WHO) Regional Office for Africa, Brazzaville, Republic of Congo; [2]CubecXpert, Quebec, QC, Canada; [3]Department of Epidemiology and Public Health, Swiss Tropical and Public Health Institute, Basel, Switzerland; [4]Health Information Systems, World Health Organization (WHO) Regional Office for Africa, InterCountry Support team for Eastern and Southern Africa Harare, Zimbabwe;[5]World Health Organization (WHO) Regional Office for Africa, Brazzaville, Republic of Congo;

Email addresses for all authors

HKK, kiprutohi@who.int
ABC, ali.bencharif@gmail.com
DOA, doriso28@gmail.com
SS, sys@who.int
HCK, karamagih@gmail.com
OT, oyeladet@who.int
BD, drotib@who.int

***Corresponding author:** Dr. Humphrey Cyprian KARAMAGI
World Health Organization (WHO) Regional Office for Africa, Brazzaville, Republic of Congo | karamagih@who.int

Word Count: 2,753/4,000 words | **Abstract:** 300/300 words | **Figures:** 0 | **Tables:** 1 | **References:** 37.

For peer review only - http://bmjopen.bmj.com/site/about/guidelines.xhtml

Abstract

Introduction: Addressing health inequities in health service coverage is a global priority, especially with the resurgence of interest in universal health coverage. However, in Africa, which has the lowest health service coverage index, there is limited information on the progress of countries in addressing health inequalities related to universal health coverage. Thus, we seek to map the evidence on the state of inequality in health service coverage and its constituent components in Africa.

Methods and analysis: We will conduct a scoping review following the Joanna Briggs Institute (JBI) Manual for Evidence Synthesis. We preregistered this protocol with the Open Science Framework (OSF) on July 26, 2022 (https://osf.io/zd5bt). We will consider any empirical research from any study design that assesses health equity in relation to health service coverage in Africa. We will search MEDLINE, Embase, Web of Science, CINAHL, PsycINFO, and Cochrane Library from their inception onwards. We will also hand-search Google and Global Index Medicus, and screen the reference lists of included or relevant studies. We will evaluate studies for eligibility and extract data from included studies using prepiloted and standardized forms. We will further extract a core set of indicators which are disaggregated by different equity stratifiers. We will summarize data using a narrative approach involving thematic syntheses and descriptive statistics. We will report our findings according to the Preferred Reporting Items for Systematic Reviews and Meta-Analysis (PRISMA) extension for Scoping Reviews checklist.

Ethics and dissemination: Ethical approval is not required as primary data will not be collected. This work will contribute to identifying knowledge gaps in the evidence of equity in health service coverage in Africa, and propose strategies that could help overcome current challenges in universal health coverage. We will disseminate our findings to knowledge users through a publication in a peer-reviewed journal and organization of workshops.

Keywords: Equity, inequity, inequality, disparities, deprivation, PROGRESS-Plus, universal health coverage, Africa, scoping review.

For peer review only

For peer review only - http://bmjopen.bmj.com/site/about/guidelines.xhtml

Strengths and limitations of this study

1. Addressing health inequities is a global priority, especially with the resurgence of interest in universal health coverage in Africa
2. This review will fill an important gap by mapping the evidence that exists on the progress towards equitable service coverage across Africa, a critical component of universal health coverage
3. This review will highlight key research gaps and data for monitoring inequalities around essential health services in Africa
4. The identification of literature will be performed using a comprehensive search through searching electronic databases, and-searching relevant websites, and screening reference lists of relevant reports
5. The search terms for equity will be comprehensive to encompass all the elements of PROGRESS-Plus, but the use of the broader term of universal health coverage may limit the identification of records which focused on specific indicators for universal health coverage

For peer review only - http://bmjopen.bmj.com/site/about/guidelines.xhtml

Introduction

Attainment of good health and well-being has been prioritized as a common goal by African countries, as encapsulated in the third Sustainable Development Goal (SDG 3) established by the United Nations in 2015 [1]. Within the World Health Organization (WHO) Regional Office for Africa, countries have recognized attainment of universal health coverage as a core result needed to attain this goal - together with attainment of good health security, and coverage of health determinants [2,3]. Universal health coverage aims to ensure that all people receive the health services they need, including promotive, preventative, curative, rehabilitative and palliative care which are of sufficient quality while ensuring that individuals do not suffer from financial hardship [4]. The ability to equitably utilize essential services needed without leaving anyone behind is one of the three thrusts of universal health coverage. Equity in service utilization is therefore an integral component of universal health coverage.

WHO defines health equity as “the absence of unfair and unavoidable or remediable differences in health among population groups defined socially, economically, demographically or geographically” [5]. In principle, health inequities are systematic differences that are socially produced, and put groups disadvantaged already at further disadvantage related to their health [6]. These inequities are considered to be key contributors to the inequalities seen in health outcomes. Monitoring of equalities is therefore considered a vital and pragmatic means to tracking and targeting inequities. The 2021 Global Monitoring report on universal health coverage showed that inequalities persist within and across countries [4]. However, consolidated evidence on inequalities,

For peer review only - http://bmjopen.bmj.com/site/about/guidelines.xhtml

especially across the range of services needed for universal health coverage attainment is lacking. At present, most evidence is either focused on specific services, or drivers of inequality. Furthermore, different dimensions of inequality may call for different strategies to address them [7].

Health inequalities are multidimensional and complex, with different drivers acting and interacting with each other and other contextual influences to produce the emergent effects on essential services utilization. Monitoring the different drivers within a similar framework is therefore crucial. The WHO commission on social determinants of health in 2005 emphasized the role of structural mechanisms that interplay between context, socio-economic position to generate and reinforce class divisions that define individual socioeconomic position within hierarchies of power, prestige and access to resources [5]. The commission identified the most important stratifiers for equality as income, education, occupation, social or class, gender, and racial or ethnicity. In sub-Saharan Africa, other studies have also recognized the need to include historical and cultural context, which underlies causal factors for the social determinants of health in the region [8,9]. Developing methodological approaches and assessing equitable access to service coverage for the different indicators of universal health coverage, an essential step in developing interventions to tackle these inequities.

Few reviews have specifically examined equity within the universal health coverage context. One narrative review published in 2014 [10] found that a key area in regards to health equity was access to quality services and specialized clinical services. However, among the 18 studies and reports included in the review, none of the country or regional

For peer review only - http://bmjopen.bmj.com/site/about/guidelines.xhtml

level articles were from Africa. Other reviews have focused primarily on specific services such as those for maternal health services [11,12].

Although health service coverage in sub-Saharan Africa is relatively low compared to other regions, existing disparities in terms of wealth and education may exacerbate the extent of service coverage among different populations. In this review, we seek to consolidate evidence on the state of inequality in the progress towards universal health coverage service coverage and/or its constituent components in Africa. The specific objectives of the review are: 1) Map the methodological approaches used in assessing health inequalities in relation to universal health coverage service coverage or its constituent components; 2) Characterize the current evidence of progress towards equity in universal health coverage service coverage or its constituent components; 3) Identify knowledge gaps in the existing evidence of progress towards equity in service coverage and propose strategies that could help overcome current challenges; 4) Document effective strategies been used to tackling the different drivers of inequalities in contributing to universal health coverage service coverage or its constituent components; and 5) Identify challenges related to addressing health equities in the region.

Methods and analysis

Design

We will conduct a scoping review following the methodology recommended in the Joanna Briggs Institute (JBI) Manual for Evidence Synthesis [13]. This methodology is based on the Arksey and O'Malley framework [14] and an enhanced version developed by Levac and colleagues [15]. Scoping reviews are defined as "a type of evidence

For peer review only - http://bmjopen.bmj.com/site/about/guidelines.xhtml

synthesis that aims to systematically identify and map the breadth of evidence available on a particular topic, field, concept, or issue, often irrespective of source (i.e., primary research, reviews, non-empirical evidence) within or across particular contexts" [16]. A scoping was selected as we aim to outline the evidence in the area of interest and identify knowledge gaps. This protocol has been registered with the Open Science Framework (OSF) on July 26, 2022 (identifier: https://osf.io/zd5bt). We will report this review according to the Preferred Reporting Items for Systematic Reviews and Meta-Analysis (PRISMA) extension for Scoping Reviews (PRISMA-ScR) checklist [17]. In this protocol, the noun "report" refers to a document (paper or electronic) supplying information about a study, and the noun "record" refers to the title or abstract of a report indexed in a database or website [18]. We report the content for this scoping review protocol using the PRISMA Protocol checklist **(Appendix 1)** [19].

Eligibility criteria

Following the Joanna Briggs Institute Manual for Evidence Synthesis [13], we will use the following eligibility criteria:

- **Participants:** We will consider any studies involving individuals, communities, or organizations, whose health needs are supposed to be addressed within a specific health system context in Africa. No restrictions based on socio-demographic factors (e.g., sex, age, ethnicity) or health conditions (e.g. comorbidities) will be applied. We will consider any countries or geographic regions in the African continent such as the 47 Member States of the WHO Regional Office for Africa, the Maghreb, and all other African regions. Global studies which include both an African region and other regions from other

For peer review only - http://bmjopen.bmj.com/site/about/guidelines.xhtml

continents (e.g., Europe, America) will be considered. We will exclude studies involving only regions or countries outside the African geographic region.

- **Concept:** We will consider any studies assessing health equity using index (e.g., concentration index) or an equity stratifier. By health equity, we mean the absence of systematic health disparities or differences among different individuals, groups, communities, or regions that are unfair and avoidable. We will only consider equity in health care coverage which refers to the access to or use of health services (i.e., equal treatment for equal need) [20,21]. To identify equity stratifiers, we will adopt the Cochrane and Campbell Equity Methods group framework of PROGRESS-Plus (place of residence, race/ethnicity/culture, occupation, gender, religion, education, socioeconomic status, and social capital). This framework includes social factors that can influence health disparities and the "Plus" includes other factors in which health inequalities may exist such as age, disability, and sexual orientation. Differences in service coverage across the PROGRESS-Plus will be considered as differences in health equity if studies provided justification that differences were unfair [22]. We will exclude studies focusing on equity in health financing or financial protection. However, studies which assess financial hardship as a driver of inequalities of health service will be included.

- **Context:** We will consider any studies monitoring progress towards universal health coverage, which means that all individuals and communities receive the health services they need. As recommended by the WHO Regional Office for Africa, we will consider 13 essential health services within three components of

For peer review only - http://bmjopen.bmj.com/site/about/guidelines.xhtml

service coverage in: 1) reproductive, maternal, newborn and child health (family planning, antenatal and delivery care, full child immunization, and health-seeking behaviour for pneumonia), 2) infectious diseases (tuberculosis treatment, HIV antiretroviral treatment, use of insecticide-treated bed nets for malaria prevention, adequate sanitation, and neglected tropical diseases treatment and care); and 3) non-communicable diseases (prevention and treatment of raised blood pressure, prevention and treatment of raised blood glucose, cervical cancer screening, and tobacco (non-)smoking) (**Appendix 2**). These health services are included in a framework of indicators developed by WHO and World Bank to track the progress of universal health coverage. We will consider 12 essential health services related to access of essential quality health services [23], to which we will add neglected tropical diseases treatment and care, which is important in the context of Africa. We will also consider any overall universal health coverage service coverage index or any universal coverage service coverage subindex on those components (e.g., universal health coverage service coverage subindex on infectious diseases). We will exclude indicators or indices related to basic hospital access, health worker density, access to essential medicines, or health security.

- **Types of sources:** We will consider any empirical studies using qualitative, quantitative, or mixed methods designs. Knowledge syntheses (e.g., systematic reviews), experimental, quasi-experimental, and observational designs will be considered. No restrictions based on language of publication or publication status will be applied. We excluded any studies published before 2005, when Universal

For peer review only

For peer review only - http://bmjopen.bmj.com/site/about/guidelines.xhtml

Coverage was cited in a World Health Assembly resolution [24]. We will also exclude any retracted publication, conference abstract, study protocol, and any editorial material. The latter refers to any report that gives the opinions of a person, group, or organization (e.g., editorials, commentaries, and letters).

In other words, we will consider any empirical research from any study design that measures health equity in relation to universal health service coverage in Africa (**Table 1**).

Table 1. Criteria for considering studies for this review

Criteria	Inclusion	Exclusion
**Type of sources**	Empirical studies using a quantitative, qualitative, or mixed methods design and published from 2005:<br>□ Original studies<br>□ Conference articles<br>□ Knowledge syntheses<br>□ Government documents	⊗ Editorial materials (e.g., commentary, letter, editorials)<br>⊗ Conference abstracts<br>⊗ Protocols<br>⊗ Retracted publications<br>⊗ Records published before 2005
**Participants**	Individuals, communities, or organizations involved in receipt of health care services within a health system context in Africa	⊗ Any countries or regions outside the African geographic region
**Concept**	Studies focusing on equity in health care services using an index (e.g., concentration index) or one of the following equity stratifier:<br>□ Place of residence (e.g., rural, urban)<br>□ Race, ethnicity, or culture<br>□ Occupation<br>□ Gender or sex<br>□ Religion<br>□ Education<br>□ Socioeconomic status<br>□ Social capital or resources<br>□ Any other factors in which health inequalities may exist (e.g., age, disability, and sexual orientation).	⊗ Studies focusing on equity in health financing<br>⊗ Studies focusing on equity in financial protection<br>⊗ Studies that did not include an equity stratifier
**Context**	Studies monitoring at least one indicator related to the following 13 essential health services (Appendix 2):<br>□ Family planning<br>□ Antenatal and delivery care<br>□ Child immunization<br>□ Health-seeking behaviour for pneumonia<br>□ Tuberculosis treatment	⊗ Studies focusing on basic hospital access<br>⊗ Studies focusing on health worker density<br>⊗ Studies focusing on access to essential medicines<br>⊗ Studies focusing on health security<br>⊗ Studies that did not include any of the 13 essential health services

For peer review only - http://bmjopen.bmj.com/site/about/guidelines.xhtml

	• Human immunodeficiency virus (HIV) antiretroviral treatment<br>• Use of insecticide-treated bed nets for malaria prevention<br>• Adequate sanitation<br>• Neglected tropical diseases treatment and care<br>• Prevention and treatment of raised blood pressure<br>• Prevention and treatment of raised blood glucose<br>• Cervical cancer screening<br>• Tobacco (non-)smoking	

Literature search

We will perform a comprehensive search to identify records through both electronic databases and secondary searches using other relevant sources. No restrictions regarding date of publication, language, place of publication, or type of reports will be applied to our search strategy.

We will search MEDLINE, Embase, Web of Science, CINAHL, PsycINFO, and the Cochrane Library from their dates of inception onwards. We will perform the preliminary search strategy in Ovid MEDLINE following appropriate design principles [25]. This preliminary search strategy will be reviewed by an information specialist and our core team of international experts in health equity, universal health coverage, health information systems, or knowledge syntheses from Africa. The search terms will be adapted to the above-mentioned databases. The search terms will be based on previous works to reflect three concepts: 1) equity, 2) universal health coverage, and 3) African regions (**Appendix 3)**. For equity, we will adapt a validate search filter [26], by adding other terms which are relevant in the African context (e.g., sexual and gender minorities) [27–29]. For universal health coverage, we will use terms integral to universal health coverage that were previously identified with an exploratory search in Google, Google Scholar, and Abstract reviews [30]. For African geographic regions, we will adapt a

For peer review only

For peer review only - http://bmjopen.bmj.com/site/about/guidelines.xhtml

238 geographic African filter validated by the South African Cochrane Centre [31], by correcting the name "Mayotte" and adding other alternative missing African country names (e.g., "Ruanda", "Comoros", and "Cabo Verde"). We will use the list of African region names used to develop the low- and middle-income countries geographic search filter by the Cochrane Effective Practice and Organisation of Care (EPOC), in collaboration with the WHO and Campbell Collaboration [32,33].

244 In addition, we will identify other relevant articles by hand-searching other relevant sources and screening reference lists of included or relevant reports. We will search Google and Global Index Medicus. We will screen at least the first 30 results for each search, a threshold often used to analyze medical content available on websites. Results lower down the relevancy lists are often duplications of earlier results and it is rare for users to click past the third page of ten search results per page [34,35].

250 Selection of sources of evidence

251 Following completion of the search, all the records identified from electronic databases will be exported to EndNote 20, and duplicates will be removed. Screening standardized forms will be prepared in Google Sheets based on the eligibility criteria and pilot tested by the entire review team to ensure reliability in accurately selecting eligible records or reports. As suggested by Joanna Briggs Institute, a random sample of 25 records will be selected for the pilot test and reviewers will only start screening when 75% or greater agreement is achieved. We calculated inter-reviewer agreement using the weighted Cohen's kappa [36]. One reviewer will screen all remaining records and assess all potentially relevant reports to identify reports meeting the eligibility criteria. Each record or report will be screened by one single reviewer using standardized forms in Google

For peer review only - http://bmjopen.bmj.com/site/about/guidelines.xhtml

Sheets and checked by another. For each ineligible report, we will document a reason for the exclusion. Any discrepancies will be resolved through consensus or with a third reviewer.

Data charting process

We will develop a form in Google sheets in consultation with the core team to guide extraction of variables. Two reviewers will independently perform a pilot test of the form to ensure the form captures relevant data. We will extract the following information: study characteristics (e.g., title, authors, and year of publication); participants (e.g., all age groups, pregnant mothers, children under five, adults, and seniors); context (e.g., name of countries), equity dimensions (e.g., PROGRESS-Plus elements); and methodological approach used to measure equality (e.g., indices). In addition, study findings will be charted against the universal health service coverage indicators. Full charting will be completed by one reviewer and checked by another. Any discrepancies between reviewers will be resolved by discussion or with a third reviewer.

Critical appraisal

Due to the nature of our research question, we will not perform an appraisal for risk of bias or conduct quality assessment. This is consistent with the Joanna Briggs Institute Manual for Evidence Synthesis [13].

Synthesis of results

We will use both qualitative and quantitative methods to analyze the data generated. We will analyze and present a descriptive summary of characteristics of included studies along with an explanatory narrative [15]. Studies will also be mapped against the

For peer review only - http://bmjopen.bmj.com/site/about/guidelines.xhtml

appropriate universal health coverage service indicators and PROGRESS-Plus elements. In addition, the study findings and discussions will be analyzed using content analysis to develop codes and themes that emerge from the data. We will use the PRISMA 2020 flowchart to describe the process of report selection [18].

Patient, public, or knowledge user involvement

Patients, and the public will not be involved in the design, conduct, or parting of this scoping review. However, we will use an integrated knowledge translation approach, where policy makers, clinicians, researchers, and trainees co-create research to ensure that it is relevant and useful to knowledge users and will increase the likelihood of uptake [37]. This will involve extensive participation of female and male international experts from Africa as equal members of the core team. Team members will meet weekly to discuss the progress of work during the current week. We will use a virtual teamwork space using Google Drive and communicate using Microsoft Teams. We will also conduct multidisciplinary consultations with colleagues from the WHO Regional Office for Africa to receive theoretical, conceptual, or practical insights for guiding the interpretation and dissemination of findings.

Discussion

This scoping review seeks to map the evidence on the state of inequality in the progress towards universal health service coverage and its constituent components in Africa. It will fill an important gap by providing a comprehensive body of evidence that exists on the progress towards equity in universal health coverage across Africa. First, it will be possible to map the evidence of equitable realization of coverage for the broad range of

For peer review only - http://bmjopen.bmj.com/site/about/guidelines.xhtml

essential services under universal health coverage across several dimensions of inequalities. Second, it will be able to highlight the barriers and opportunities of addressing effectively the drivers of health inequalities in Africa. Thirdly, it will be possible to demonstrate which methodological approaches are appropriate for measuring health inequalities in the African context. Lastly, it will highlight which essential services and its relevant dimensions of inequalities that have been under-researched in the literature, and may need future investigations. Thus, this review will make a critical contribution to monitoring equity in universal health coverage by promoting learning and building the evidence for investments in effective strategies to reduce health equities in Africa.

Ethics and dissemination

This scoping review will involve neither human participants nor unpublished secondary data. As such, formal ethical approval from a research ethics committee is not required. We will disseminate our results through publications in peer-reviewed journals, and a technical report for the WHO Regional Office for Africa. We will also share our reports using free public repositories such as Open Science Framework and ResearchGate.

For peer review only - http://bmjopen.bmj.com/site/about/guidelines.xhtml

List of abbreviations

PRISMA: Preferred Reporting Items for Systematic Reviews and Meta-Analysis;
PRISMA-ScR: PRISMA extension for Scoping Reviews;
PROGRESS: Place of residence, Race/ethnicity/culture, Occupation, Gender, Religion, Education, Socioeconomic status, and Social capital)
WHO: World Health Organization;

Declarations

Ethics approval and consent to participate

Not applicable.

Consent for publication

Not applicable.

Availability of data and materials

Please send all requests for study data or materials to Dr. Humphrey Cyprian KARAMAGI (karamagih@who.int).

Competing interests

Authors declare that they have no competing interests.

Funding

No direct funding was provided.

Authors' contributions

The World Health Organization (WHO) Regional Office for Africa participated in the conception of the idea of this knowledge synthesis (HCK, SS, HKK, OT, and BD). HCK, ABC, DOA, SS, and HKK participated in the design of this protocol. ABC drafted the search strategy and all authors revised it. ABC and DOA drafted this protocol. All authors revised the manuscript critically for important intellectual content, gave final approval of the version to be published and agreed to be accountable for all aspects of the knowledge synthesis.

Acknowledgements

We wish to acknowledge the World Health Organization (WHO) Regional Office for Africa for their assistance with various aspects of this work.

For peer review only

For peer review only - http://bmjopen.bmj.com/site/about/guidelines.xhtml

References

1 General Assembly. Resolution adopted by the General Assembly on 11 September 2015. New York: : United Nations 2015.

2 Karamagi HC, Tumusiime P, Titi-Ofei R, *et al.* Towards universal health coverage in the WHO African Region: assessing health system functionality, incorporating lessons from COVID-19. *BMJ Global Health* 2021;**6**:e004618. doi:10.1136/bmjgh-2020-004618

3 Karamagi HC, Ben Charif A, Kidane SN, *et al.* Investments for effective functionality of health systems towards Universal Health Coverage in Africa: A scoping review. *PLOS Global Public Health* 2022;**2**:e0001076. doi:10.1371/journal.pgph.0001076

4 World Health Organization, World Bank. Tracking Universal Health Coverage: 2021 Global Monitoring Report. Washington, DC: : World Bank 2021. https://openknowledge.worldbank.org/handle/10986/36724 (accessed 5 Jul 2022).

5 World Health Organization. A conceptual framework for action on the social determinants of health. World Health Organization 2010. https://apps.who.int/iris/handle/10665/44489 (accessed 5 Jul 2022).

6 Braveman P, Gruskin S. Defining equity in health. *J Epidemiol Community Health* 2003;**57**:254–8. doi:10.1136/jech.57.4.254

7 Hosseinpoor AR, Bergen N, Koller T, *et al.* Equity-Oriented Monitoring in the Context of Universal Health Coverage. *PLOS Medicine* 2014;**11**:e1001727. doi:10.1371/journal.pmed.1001727

8 Eshetu E, Woldesenbet S. Are there particular social determinants of health for the world's poorest countries? *Afr Health Sci* 2011;**11**:108–15.

9 Ichoku HE, Mooney G, Ataguba JE-O. Africanizing the social determinants of health: embedded structural inequalities and current health outcomes in sub-Saharan Africa. *Int J Health Serv* 2013;**43**:745–59. doi:10.2190/HS.43.4.i

10 Rodney AM, Hill PS. Achieving equity within universal health coverage: a narrative review of progress and resources for measuring success. *International Journal for Equity in Health* 2014;**13**:72. doi:10.1186/s12939-014-0072-8

11 Çalışkan Z, Kılıç D, Öztürk S, *et al.* Equity in maternal health care service utilization: a systematic review for developing countries. *Int J Public Health* 2015;**60**:815–25. doi:10.1007/s00038-015-0711-x

12 Målqvist M, Yuan B, Trygg N, *et al.* Targeted Interventions for Improved Equity in Maternal and Child Health in Low- and Middle-Income Settings: A Systematic

For peer review only

For peer review only - http://bmjopen.bmj.com/site/about/guidelines.xhtml

Review and Meta-Analysis. *PLOS ONE* 2013;**8**:e66453. doi:10.1371/journal.pone.0066453

13 Peters M, Godfrey C, McInerney P, *et al.* Chapter 11: Scoping Reviews. In: Aromataris E, Munn Z, eds. *JBI Manual for Evidence Synthesis*. JBI 2020. doi:10.46658/JBIMES-20-12

14 Arksey H, O'Malley L. Scoping studies: towards a methodological framework. *International Journal of Social Research Methodology* 2005;**8**:19–32. doi:10.1080/1364557032000119616

15 Levac D, Colquhoun H, O'Brien KK. Scoping studies: advancing the methodology. *Implementation Science* 2010;**5**:69. doi:10.1186/1748-5908-5-69

16 Munn Z, Pollock D, Khalil H, *et al.* What are scoping reviews? Providing a formal definition of scoping reviews as a type of evidence synthesis. *JBI Evidence Synthesis* 2022;**20**:950–2. doi:10.11124/JBIES-21-00483

17 Tricco AC, Lillie E, Zarin W, *et al.* PRISMA Extension for Scoping Reviews (PRISMA-ScR): Checklist and Explanation. *Ann Intern Med* 2018;**169**:467–73. doi:10.7326/M18-0850

18 Page MJ, McKenzie JE, Bossuyt PM, *et al.* The PRISMA 2020 statement: an updated guideline for reporting systematic reviews. *BMJ* 2021;**372**:n71. doi:10.1136/bmj.n71

19 Moher D, Shamseer L, Clarke M, *et al.* Preferred reporting items for systematic review and meta-analysis protocols (PRISMA-P) 2015 statement. *Syst Rev* 2015;**4**:1. doi:10.1186/2046-4053-4-1

20 Cohen AB, Grogan CM, Horwitt JN. The Many Roads toward Achieving Health Equity. *Journal of Health Politics, Policy and Law* 2017;**42**:739–48. doi:10.1215/03616878-3940414

21 Paul E, Deville C, Bodson O, *et al.* How is equity approached in universal health coverage? An analysis of global and country policy documents in Benin and Senegal. *International Journal for Equity in Health* 2019;**18**:195. doi:10.1186/s12939-019-1089-9

22 Cochrane Methods Equity. PROGRESS-Plus. https://methods.cochrane.org/equity/projects/evidence-equity/progress-plus (accessed 18 Jul 2022).

23 World Health Organization (WHO). Universal health coverage (UHC). https://www.who.int/news-room/fact-sheets/detail/universal-health-coverage-(uhc) (accessed 12 Sep 2022).

24 World Health Assembly 58. Social health insurance: sustainable health financing, universal coverage and social health insurance: report by the Secretariat. World

For peer review only

For peer review only - http://bmjopen.bmj.com/site/about/guidelines.xhtml

Health Organization 2005. https://apps.who.int/iris/handle/10665/20302 (accessed 3 Aug 2022).

25 MacFarlane A, Russell-Rose T, Shokraneh F. Search strategy formulation for systematic reviews: Issues, challenges and opportunities. *Intelligent Systems with Applications* 2022;**15**:200091. doi:10.1016/j.iswa.2022.200091

26 Hosking J, Macmillan A, Jones R, *et al.* Searching for health equity: validation of a search filter for ethnic and socioeconomic inequalities in transport. *Systematic Reviews* 2019;**8**:94. doi:10.1186/s13643-019-1009-5

27 Parker R, Wanner A, Foster M, *et al.* Design and validation of a search filter for LGBTQ+ populations. In: *25th Cochrane Colloquium*. Edinburgh, UK: 2018. 16–8.https://doi.org/10.1002/14651858.CD201801

28 Rizvi A, Lawson DO, Young T, *et al.* Guidance relevant to the reporting of health equity in observational research: a scoping review protocol. *BMJ Open* 2022;**12**:e056875. doi:10.1136/bmjopen-2021-056875

29 Prady SL, Uphoff EP, Power M, *et al.* Development and validation of a search filter to identify equity-focused studies: reducing the number needed to screen. *BMC Medical Research Methodology* 2018;**18**:106. doi:10.1186/s12874-018-0567-x

30 Rizvi SS, Douglas R, Williams OD, *et al.* The political economy of universal health coverage: a systematic narrative review. *Health Policy and Planning* 2020;**35**:364–72. doi:10.1093/heapol/czz171

31 Pienaar E, Grobler L, Busgeeth K, *et al.* Developing a geographic search filter to identify randomised controlled trials in Africa: finding the optimal balance between sensitivity and precision. *Health Info Libr J* 2011;**28**:210–5. doi:10.1111/j.1471-1842.2011.00936.x

32 Cochrane Effective Practice and Organisation of Care (EPOC). Low- and middle-income country (LMIC) filters. 2020.https://epoc.cochrane.org/lmic-filters (accessed 25 Nov 2021).

33 Sutton A, Campbell F. The ScHARR LMIC filter: Adapting a low- and middle-income countries geographic search filter to identify studies on preterm birth prevention and management. *Res Synth Methods* 2022;**13**:447–56. doi:10.1002/jrsm.1552

34 Hargrave DR, Hargrave UA, Bouffet E. Quality of health information on the Internet in pediatric neuro-oncology. *Neuro-oncol* 2006;**8**:175–82. doi:10.1215/15228517-2005-008

35 van der Marel S, Duijvestein M, Hardwick JC, *et al.* Quality of web-based information on inflammatory bowel diseases. *Inflamm Bowel Dis* 2009;**15**:1891–6. doi:10.1002/ibd.20976

For peer review only - http://bmjopen.bmj.com/site/about/guidelines.xhtml

36 Cohen J. Weighted kappa: Nominal scale agreement provision for scaled disagreement or partial credit. *Psychological Bulletin* 1968;**70**:213–20. doi:10.1037/h0026256

37 Canadian Institutes of Health Research (CIHR). Guide to Knowledge Translation Planning at CIHR: Integrated and End-of-Grant Approaches. 2012.http://www.cihr-irsc.gc.ca/e/45321.html#a5 (accessed 28 Feb 2018).

For peer review only

For peer review only - http://bmjopen.bmj.com/site/about/guidelines.xhtml

Tables

Table 1. Criteria for considering studies for this review

Additional files

Appendix 1. The the Preferred Reporting Items for Systematic Reviews and Meta-Analysis Protocol (PRISMA-P) checklist

Appendix 2. Health service coverage indicators to be included for measuring progress towards universal health coverage in this review

Appendix 3. Search strategy for Ovid MEDLINE

For peer review only

For peer review only - http://bmjopen.bmj.com/site/about/guidelines.xhtml

PRISMA-P 2015 Checklist

This checklist has been adapted for use with protocol submissions to *Systematic Reviews* from Table 3 in Moher D et al: Preferred reporting items for systematic review and meta-analysis protocols (PRISMA-P) 2015 statement. *Systematic Reviews* 2015 **4**:1

Section/topic	#	Checklist item	Information reported		Line number(s)
			Yes	No	
**ADMINISTRATIVE INFORMATION**					
**Title**					
Identification	1a	Identify the report as a protocol of a systematic review	☒	☐	2
Update	1b	If the protocol is for an update of a previous systematic review, identify as such	☐	☐	Not applicable
**Registration**	2	If registered, provide the name of the registry (e.g., PROSPERO) and registration number in the Abstract	☒	☐	41
**Authors**					
Contact	3a	Provide name, institutional affiliation, and e-mail address of all protocol authors; provide physical mailing address of corresponding author	☒	☐	6-27
Contributions	3b	Describe contributions of protocol authors and identify the guarantor of the review	☒	☐	345-352
**Amendments**	4	If the protocol represents an amendment of a previously completed or published protocol, identify as such and list changes; otherwise, state plan for documenting important protocol amendments	☐	☐	Not applicable
**Support**					
Sources	5a	Indicate sources of financial or other support for the review	☒	☐	342-343
Sponsor	5b	Provide name for the review funder and/or sponsor	☒	☐	342-343
Role of sponsor/funder	5c	Describe roles of funder(s), sponsor(s), and/or institution(s), if any, in developing the protocol	☒	☐	342-343
**INTRODUCTION**					
**Rationale**	6	Describe the rationale for the review in the context of what is already known	☒	☐	76-125
**Objectives**	7	Provide an explicit statement of the question(s) the review will address with reference to participants, interventions, comparators, and outcomes (PICO)	☒	☐	125-136

For peer review only

BioMed Central
The Open Access Publisher

Section/topic	#	Checklist item	Information reported		Line number(s)
			Yes	No	
**METHODS**					
**Eligibility criteria**	8	Specify the study characteristics (e.g., PICO, study design, setting, time frame) and report characteristics (e.g., years considered, language, publication status) to be used as criteria for eligibility for the review	☒	☐	155-218
**Information sources**	9	Describe all intended information sources (e.g., electronic databases, contact with study authors, trial registers, or other grey literature sources) with planned dates of coverage	☒	☐	219-248
**Search strategy**	10	Present draft of search strategy to be used for at least one electronic database, including planned limits, such that it could be repeated	☒	☐	219-248
STUDY RECORDS					
Data management	11a	Describe the mechanism(s) that will be used to manage records and data throughout the review	☒	☐	138-262
Selection process	11b	State the process that will be used for selecting studies (e.g., two independent reviewers) through each phase of the review (i.e., screening, eligibility, and inclusion in meta-analysis)	☒	☐	249-262
Data collection process	11c	Describe planned method of extracting data from reports (e.g., piloting forms, done independently, in duplicate), any processes for obtaining and confirming data from investigators	☒	☐	263-273
**Data items**	12	List and define all variables for which data will be sought (e.g., PICO items, funding sources), any pre-planned data assumptions and simplifications	☒	☐	263-273
**Outcomes and prioritization**	13	List and define all outcomes for which data will be sought, including prioritization of main and additional outcomes, with rationale	☒	☐	263-273
**Risk of bias in individual studies**	14	Describe anticipated methods for assessing risk of bias of individual studies, including whether this will be done at the outcome or study level, or both; state how this information will be used in data synthesis	☒	☐	274-277
DATA					
**Synthesis**	15a	Describe criteria under which study data will be quantitatively synthesized	☒	☐	278-286
	15b	If data are appropriate for quantitative synthesis, describe planned summary measures, methods of handling data, and methods of combining data from studies, including any planned exploration of consistency (e.g., I^2, Kendall's tau)	☒	☐	278-286
	15c	Describe any proposed additional analyses (e.g., sensitivity or subgroup analyses, meta-regression)	☒	☐	278-286
	15d	If quantitative synthesis is not appropriate, describe the type of summary planned	☒	☐	278-286

For peer review only

BioMed Central The Open Access Publisher

Section/topic	#	Checklist item	Information reported		Line number(s)
			Yes	No	
**Meta-bias(es)**	16	Specify any planned assessment of meta-bias(es) (e.g., publication bias across studies, selective reporting within studies)	☒	☐	274-286
**Confidence in cumulative evidence**	17	Describe how the strength of the body of evidence will be assessed (e.g., GRADE)	☒	☐	278-286

For peer review only

BioMed Central
The Open Access Publisher

Appendix 2: Health service coverage indicators to be included for measuring progress towards universal health coverage in this review

**Indicators for measuring progress towards reproductive, maternal, newborn and child health (RMNCH)**
**1. Family planning** (e.g., proportion of births attended by skilled health personnel, demand satisfied with modern contraception methods in women aged 15-49 years)
**2. Antenatal and delivery care** (e.g., proportion of visits)
**3. Child immunization** (e.g., proportion of children aged 1 year who have received three doses of diphtheria, tetanus, and pertussis vaccine (DPT3), measles vaccine, or bacille Calmette-Guérin (BCG) vaccine)
**4. Health-seeking behaviour for pneumonia** (e.g., proportion of children with suspected pneumonia who have sought care, proportion of children with oral rehydration therapy for diarrheal diseases, proportion of children who were breastfed within the first hour of birth)
**Indicators for measuring progress towards infectious diseases**
**5. Tuberculosis treatment** (e.g., proportion of people who received effective tuberculosis treatment)
**6. Human immunodeficiency virus (HIV) antiretroviral treatment** (e.g., proportion of people with HIV receiving antiretroviral treatment)
**7. Use of insecticide-treated bed nets for malaria prevention** (e.g., proportion of households receiving insecticide-treated bed nets, percentage of people sleeping under insecticide-treated nets)
**8. Adequate sanitation** (e.g., proportion of rural hospitals with basic sanitation)
**9. Neglected tropical diseases treatment and care (**e.g., number of people required treatment for any of the neglected tropical diseases). Neglected tropical diseases are a diverse group of 20 conditions including: Buruli ulcer, Chagas disease, dengue and chikungunya, dracunculiasis (Guinea-worm disease), echinococcosis, foodborne trematodiases, human African trypanosomiasis (sleeping sickness), leishmaniasis, leprosy (Hansen's disease), lymphatic filariasis, mycetoma, chromoblastomycosis and other deep mycoses, onchocerciasis (river blindness), rabies, scabies and other ectoparasitoses, schistosomiasis, soil-transmitted helminthiases, snakebite envenoming, taeniasis/cysticercosis, trachoma, and yaws and other endemic treponematoses.
**Indicators for measuring progress towards non-communicable diseases**
**10. Prevention and treatment of raised blood pressure** (e.g.,prevalence of raised blood pressure regardless of treatment status)
**11. Prevention and treatment of raised blood glucose** (e.g., mean fasting plasma glucose)
**12. Cervical cancer screening** (e.g., prevalence of cervical cancer screening at aged 30-49)
**13. Tobacco (non-)smoking** (e.g., prevalence of tobacco use among adults aged at least 15 years)

For peer review only - http://bmjopen.bmj.com/site/about/guidelines.xhtml

Appendix 3. Search strategy for Ovid MEDLINE

MEDLINE via Ovid (searched on August 29, 2022; Period: 1946 - August 26, 2022)

Concept		Keyword	umber	Result
**Equity**	**Controlled Vocabulary**	exp "gender identity"/ or exp "sexual and gender minorities"/ or exp "disabled persons"/ or exp geriatrics/ or "health equity"/ or "health inequities"/ or "healthcare disparities"/ or "health status disparities"/ or "health services accessibility"/ or "sex factors"/ or women/ or men/ or "residence characteristics"/ or "urban population"/ or "rural population"/ or "cultural characteristics"/ or "cultural diversity"/ or religion/ or "social discrimination"/ or "socioeconomic factors"/ or "vulnerable populations"/ or "social class"/ or "minority groups"/ or "ethnic and racial minorities"/ or "educational status"/ or unemployment/	1	884,458
	**Free Text**	(equit* or inequit* or inequalit* or disparit* or equalit* or deprivation or gini or "concentration index" or ((health* or medical) adj3 (availabl* or access*)) or (gender adj (difference? or identit* or role?)) or (sex adj (disparit* or difference? or role?)) or ((wom#n* or m#n*) adj role?) or "sexual minorit*" or "gender minorit*" or LGBT* or 2SLGBT* or urban or rural or ethnic* or religi* or ((social* or socio-economic or socioeconomic or economic or structural or material) adj3 (advantage* or disadvantage* or exclude* or exclusion or include* or inclusion or status or position or gradient* or hierarch* or class* or determinant*)) or (health adj3 (gap* or gradient* or hierarch*)) or SES or SEP or sociodemographic* or socio-demographic* or income or wealth* or poverty or "educational status" or "educational level?" or "level? of education" or "educational attainment" or ((well or better or higher or worse or less) adj (educated)) or unemploy* or "home owner*" or tenure or affluen* or "well off" or "better off" or "worse off").ti,ab,kw,kf	2	1,197,912
	**All**	1 or 2	3	1,739,958
**Universal health coverage**	**Controlled Vocabulary**	"universal health care"/	4	243
	**Free Text**	(UHC or (universal adj2 (coverage or health* or care or access))).ti,ab,kw,kf	5	12,120
	**All**	4 or 5	6	12,186
**Africa**	**Controlled Vocabulary**	exp africa/ not ("guinea pig" or "guinea pigs" or "aspergillus niger").ti,ab,kw,kf,cp,in	7	308,111
	**Free Text**	((africa* or algeria or algerie or angola or benin or dahomey or botswana or bechuanaland or "burkina faso" or "burkina fasso" or "upper volta" or "haute volta" or burundi or urundi or cameroon or cameroun or "canary islands" or "cape verde" or cabo verde or "ubangi shari" or chad or tchad or comoros or "comoro islands" or comores or mayotte or congo or zaire or djibouti or "french somaliland" or egypt or "united arab republic" or eritrea or eswatini or swaziland or ethiopia or gabon or "gabonese republic" or gambia or ghana or "gold coast" or guinea or "ivory coast" or "cote d?ivoire" or "cote d’ ivoire" or kenya or lesotho or basutoland or liberia or libya or libia or jamahiriya or jamahiryia or madagascar or "malagasy republic" or malawi or nyasaland or mali or mauritania or mauritius or morocco or ifni or mozambique or "portuguese east africa" or mocambique or namibia or niger or nigeria or principe or "sao tome" or reunion or rwanda or ruanda or senegal or seychelles or "sierra leone" or somalia or "south africa" or "st helena" or sudan or tanzania or tanganyika or togo or "togolese republic" or tunisia or tunisie or uganda or "western sahara" or zambia or zimbabwe or "indian ocean islands" or sahara or magreb or magrib) not ("guinea pig" or "guinea pigs" or "aspergillus niger")).ti,ab,kw,kf,cp,in	8	885,575
	**All**	7 or 8	9	921,268
**Total result**		**3 AND 6 AND 9**	**10**	**1,924**

For peer review only

For peer review only - http://bmjopen.bmj.com/site/about/guidelines.xhtml

Embase via Elsevier (searched on August 29, 2022)

Concept		Keyword	umber	Result
**Equity**	**Controlled Vocabulary**	'gender identity'/exp or 'sexual and gender minority'/exp or 'disabled person'/exp or 'disabled person'/exp or 'health equity'/de or 'health disparity'/de or 'health care access'/de or 'health care availability'/de or 'sex difference'/de or 'female'/de or 'male'/de or 'residence characteristics'/de or 'urban population'/de or 'rural population'/de or 'cultural factor'/de or 'cultural diversity'/de or 'religion'/de or 'social discrimination'/de or 'socioeconomics'/de or 'vulnerable population'/de or 'disadvantaged population'/de or 'social class'/de or 'minority group'/de or 'ethnic group'/de or 'educational status'/de or 'unemployment'/de	#1	15,833,425
	**Free Text**	(equit* or inequit* or inequalit* or disparit* or equalit* or deprivation or gini or "concentration index" or ((health* or medical) NEAR/3 (availabl* or access*)) or (gender NEXT/1 (difference$ or identit* or role$)) or (sex NEXT/1 (disparit* or difference$ or role$)) or ((wom?n* or m?n*) NEXT/1 role$) or "sexual minorit*" or "gender minorit*" or LGBT* or 2SLGBT* or urban or rural or ethnic* or religi* or ((social* or socio-economic or socioeconomic or economic or structural or material) NEAR/3 (advantage* or disadvantage* or exclude* or exclusion or include* or inclusion or status or position or gradient* or hierarch* or class* or determinant*)) or (health NEAR/3 (gap* or gradient* or hierarch*)) or SES or SEP or sociodemographic* or socio-demographic* or income or wealth* or poverty or "educational status" or "educational level$" or "level$ of education" or "educational attainment" or ((well or better or higher or worse or less) NEXT/1 (educated)) or unemploy* or "home owner*" or tenure or affluen* or "well off" or "better off" or "worse off"):ti,ab,kw	#2	1,527,2697
	**All**	1 or 2	#3	16,237,327
**Universal health coverage**	**Controlled Vocabulary**	'universal health care'/de	#4	600
	**Free Text**	(UHC or (universal NEAR/2 (coverage or health* or care or access))):ti,ab,kw	#5	14,728
	**All**	4 or 5	#6	14,913
**Africa**	**Controlled Vocabulary**	'africa'/exp not ("guinea pig" or "guinea pigs" or "aspergillus niger"):ti,ab,kw,ca,ad,ff	#7	397,519
	**Free Text**	((africa* or algeria or algerie or angola or benin or dahomey or botswana or bechuanaland or "burkina faso" or "burkina fasso" or "upper volta" or "haute volta" or burundi or urundi or cameroon or cameroun or "canary islands" or "cape verde" or cabo verde or "ubangi shari" or chad or tchad or comoros or "comoro islands" or comores or mayotte or congo or zaire or djibouti or "french somaliland" or egypt or "united arab republic" or eritrea or eswatini or swaziland or ethiopia or gabon or "gabonese republic" or gambia or ghana or "gold coast" or guinea or "ivory coast" or "cote d$ivoire" or "cote d? ivoire" or kenya or lesotho or basutoland or liberia or libya or libia or jamahiriya or jamahiryia or madagascar or "malagasy republic" or malawi or nyasaland or mali or mauritania or mauritius or morocco or ifni or mozambique or "portuguese east africa" or mocambique or namibia or niger or nigeria or principe or "sao tome" or reunion or rwanda or ruanda or senegal or seychelles or "sierra leone" or somalia or "south africa" or "st helena" or sudan or tanzania or tanganyika or togo or "togolese republic" or tunisia or tunisie or uganda or "western sahara" or zambia or zimbabwe or "indian ocean islands" or sahara or magreb or magrib) not ("guinea pig" or "guinea pigs" or "aspergillus niger")):ti,ab,kw,ca,ad,ff	#8	859,620
	**All**	7 or 8	#9	960,563
**Total result**		**#3 AND #6 AND #9**	**#10**	2,586

For peer review only - http://bmjopen.bmj.com/site/about/guidelines.xhtml

Web of Science (searched on August 29, 2022)

Concept		Keyword	umber	Result
**Equity**	**Free Text**	TS=(equit* or inequit* or inequalit* or disparit* or equalit* or deprivation or gini or "concentration index" or ((health* or medical) NEAR/2 (availabl* or access*)) or (gender NEAR/0 (difference$ or identit* or role$)) or (sex NEAR/0 (disparit* or difference$ or role$)) or ((wom?n* or man* or men*) NEAR/0 role$) or "sexual minorit*" or "gender minorit*" or LGBT* or 2SLGBT* or urban or rural or ethnic* or religi* or ((social* or socio-economic or socioeconomic or economic or structural or material) NEAR/2 (advantage* or disadvantage* or exclude* or exclusion or include* or inclusion or status or position or gradient* or hierarch* or class* or determinant*)) or (health NEAR/2 (gap* or gradient* or hierarch*)) or SES or SEP or sociodemographic* or socio-demographic* or income or wealth* or poverty or "educational status" or "educational level$" or "level$ of education" or "educational attainment" or ((well or better or higher or worse or less) NEAR/0 (educated)) or unemploy* or "home owner*" or tenure or affluen* or "well off" or "better off" or "worse off")	#1	3,056,690
**Universal health coverage**	**Free Text**	TS=(UHC or (universal NEAR/1 (coverage or health* or care or access)))	#2	15,460
**Africa**	**Free Text**	TS=((africa* or algeria or algerie or angola or benin or dahomey or botswana or bechuanaland or "burkina faso" or "burkina fasso" or "upper volta" or "haute volta" or burundi or urundi or cameroon or cameroun or "canary islands" or "cape verde" or cabo verde or "ubangi shari" or chad or tchad or comoros or "comoro islands" or comores or mayotte or congo or zaire or djibouti or "french somaliland" or egypt or "united arab republic" or eritrea or eswatini or swaziland or ethiopia or gabon or "gabonese republic" or gambia or ghana or "gold coast" or guinea or "ivory coast" or "cote d$ivoire" or "cote d’ ivoire" or kenya or lesotho or basutoland or liberia or libya or libia or jamahiriya or jamahiryia or madagascar or "malagasy republic" or malawi or nyasaland or mali or mauritania or mauritius or morocco or ifni or mozambique or "portuguese east africa" or mocambique or namibia or niger or nigeria or principe or "sao tome" or reunion or rwanda or ruanda or senegal or seychelles or "sierra leone" or somalia or "south africa" or "st helena" or sudan or tanzania or tanganyika or togo or "togolese republic" or tunisia or tunisie or uganda or "western sahara" or zambia or zimbabwe or "indian ocean islands" or sahara or magreb or magrib) not ("guinea pig" or "guinea pigs" or "aspergillus niger")) or<br>CU=((africa* or algeria or algerie or angola or benin or dahomey or botswana or bechuanaland or "burkina faso" or "burkina fasso" or "upper volta" or "haute volta" or burundi or urundi or cameroon or cameroun or "canary islands" or "cape verde" or cabo verde or "ubangi shari" or chad or tchad or comoros or "comoro islands" or comores or mayotte or congo or zaire or djibouti or "french somaliland" or egypt or "united arab republic" or eritrea or eswatini or swaziland or ethiopia or gabon or "gabonese republic" or gambia or ghana or "gold coast" or guinea or "ivory coast" or "cote d$ivoire" or "cote d’ ivoire" or kenya or lesotho or basutoland or liberia or libya or libia or jamahiriya or jamahiryia or madagascar or "malagasy republic" or malawi or nyasaland or mali or mauritania or mauritius or morocco or ifni or mozambique or "portuguese east africa" or mocambique or namibia or niger or nigeria or principe or "sao tome" or reunion or rwanda or ruanda or senegal or seychelles or "sierra leone" or somalia or "south africa" or "st helena" or sudan or tanzania or tanganyika or togo or "togolese republic" or tunisia or tunisie or uganda or "western sahara" or zambia or zimbabwe or "indian ocean islands" or sahara or magreb or magrib) not ("guinea pig" or "guinea pigs" or "aspergillus niger")) or<br>OG=((africa* or algeria or algerie or angola or benin or dahomey or botswana or bechuanaland or "burkina faso" or "burkina fasso" or "upper volta" or "haute volta" or burundi or urundi or cameroon or cameroun or "canary islands" or "cape verde" or cabo verde or "ubangi shari" or chad or tchad or comoros or "comoro islands" or comores or mayotte or congo or zaire or djibouti or "french somaliland" or egypt or "united arab republic" or eritrea or eswatini or swaziland or ethiopia or gabon or "gabonese republic" or gambia or ghana or "gold coast" or guinea or "ivory coast" or "cote d$ivoire" or "cote d’ ivoire" or kenya or lesotho or basutoland or liberia or libya or libia or jamahiriya or jamahiryia or madagascar or "malagasy republic" or malawi	#3	2,204,863

For peer review only - http://bmjopen.bmj.com/site/about/guidelines.xhtml

		or nyasaland or mali or mauritania or mauritius or morocco or ifni or mozambique or "portuguese east africa" or mocambique or namibia or niger or nigeria or principe or "sao tome" or reunion or rwanda or ruanda or senegal or seychelles or "sierra leone" or somalia or "south africa" or "st helena" or sudan or tanzania or tanganyika or togo or "togolese republic" or tunisia or tunisie or uganda or "western sahara" or zambia or zimbabwe or "indian ocean islands" or sahara or magreb or magrib) not ("guinea pig" or "guinea pigs" or "aspergillus niger"))		
**Total result**		**#1 AND #2 AND #3**	**#4**	**2,145**

For peer review only

For peer review only - http://bmjopen.bmj.com/site/about/guidelines.xhtml

CINAHL via EBSCOhost (searched on August 19, 2022)

Concept		Keyword	umber	Result
**Equity**	**Controlled Vocabulary**	(MH "gender identity+") or (MH "sexual and gender minorities+") or (MH "persons with disabilities+") or (MH "geriatrics+") or (MH "health inequities") or (MH "healthcare disparities") or (MH "health status disparities") or (MH "health services accessibility") or (MH "sex factors") or (MH "women") or (MH "men") or (MH "residence characteristics") or (MH "urban population") or (MH "rural population") or (MH "cultural diversity") or (MH "religion and religions") or (MH "discrimination") or (MH "socioeconomic factors") or (MH "special populations") or (MH "social class") or (MH "minority groups") or (MH "ethnic groups") or (MH "educational status") or (MH "unemployment")	S1	544,616
	**Free Text**	TI (equit* or inequit* or inequalit* or disparit* or equalit* or deprivation or gini or "concentration index" or ((health* or medical) N2 (availabl* or access*)) or (gender W1 (difference# or identit* or role#)) or (sex W1 (disparit* or difference# or role#)) or ((wom?n* or m?n*) W1 role#) or "sexual minorit*" or "gender minorit*" or LGBT* or 2SLGBT* or urban or rural or ethnic* or religi* or ((social* or socio-economic or socioeconomic or economic or structural or material) N2 (advantage* or disadvantage* or exclude* or exclusion or include* or inclusion or status or position or gradient* or hierarch* or class* or determinant*)) or (health N2 (gap* or gradient* or hierarch*)) or SES or SEP or sociodemographic* or socio-demographic* or income or wealth* or poverty or "educational status" or "educational level#" or "level# of education" or "educational attainment" or ((well or better or higher or worse or less) W1 (educated)) or unemploy* or "home owner*" or tenure or affluen* or "well off" or "better off" or "worse off") OR AB (equit* or inequit* or inequalit* or disparit* or equalit* or deprivation or gini or "concentration index" or ((health* or medical) N2 (availabl* or access*)) or (gender W1 (difference# or identit* or role#)) or (sex W1 (disparit* or difference# or role#)) or ((wom?n* or m?n*) W1 role#) or "sexual minorit*" or "gender minorit*" or LGBT* or 2SLGBT* or urban or rural or ethnic* or religi* or ((social* or socio-economic or socioeconomic or economic or structural or material) N2 (advantage* or disadvantage* or exclude* or exclusion or include* or inclusion or status or position or gradient* or hierarch* or class* or determinant*)) or (health N2 (gap* or gradient* or hierarch*)) or SES or SEP or sociodemographic* or socio-demographic* or income or wealth* or poverty or "educational status" or "educational level#" or "level# of education" or "educational attainment" or ((well or better or higher or worse or less) W1 (educated)) or unemploy* or "home owner*" or tenure or affluen* or "well off" or "better off" or "worse off") OR SU (equit* or inequit* or inequalit* or disparit* or equalit* or deprivation or gini or "concentration index" or ((health* or medical) N2 (availabl* or access*)) or (gender W1 (difference# or identit* or role#)) or (sex W1 (disparit* or difference# or role#)) or ((wom?n* or m?n*) W1 role#) or "sexual minorit*" or "gender minorit*" or LGBT* or 2SLGBT* or urban or rural or ethnic* or religi* or ((social* or socio-economic or socioeconomic or economic or structural or material) N2 (advantage* or disadvantage* or exclude* or exclusion or include* or inclusion or status or position or gradient* or hierarch* or class* or determinant*)) or (health N2 (gap* or gradient* or hierarch*)) or SES or SEP or sociodemographic* or socio-demographic* or income or wealth* or poverty or "educational status" or "educational level#" or "level# of education" or "educational attainment" or ((well or better or higher or worse or less) W1 (educated)) or unemploy* or "home owner*" or tenure or affluen* or "well off" or "better off" or "worse off")	S2	654,635
	**All**	S1 or S2	S3	874,600
**Universal health coverage**	**Controlled Vocabulary**	MH "universal health care"	S4	552
	**Free Text**	TI (UHC or (universal N1 (coverage or health* or care or access))) OR AB (UHC or (universal N1 (coverage or health* or care or access))) OR SU (UHC or (universal N1 (coverage or health* or care or access)))	S5	6,489
	**All**	S4 or S5	S6	6,489

For peer review only - http://bmjopen.bmj.com/site/about/guidelines.xhtml

**Africa**	**Controlled Vocabulary**	(MH "africa+") not (TI ("guinea pig" or "guinea pigs" or "aspergillus niger") OR AB ("guinea pig" or "guinea pigs" or "aspergillus niger") OR SU ("guinea pig" or "guinea pigs" or "aspergillus niger") OR AF ("guinea pig" or "guinea pigs" or "aspergillus niger"))	S7	96,591
	**Free Text**	TI ((africa* or algeria or algerie or angola or benin or dahomey or botswana or bechuanaland or "burkina faso" or "burkina fasso" or "upper volta" or "haute volta" or burundi or urundi or cameroon or cameroun or "canary islands" or "cape verde" or cabo verde or "ubangi shari" or chad or tchad or comoros or "comoro islands" or comores or mayotte or congo or zaire or djibouti or "french somaliland" or egypt or "united arab republic" or eritrea or eswatini or swaziland or ethiopia or gabon or "gabonese republic" or gambia or ghana or "gold coast" or guinea or "ivory coast" or "cote d#ivoire" or "cote d' ivoire" or kenya or lesotho or basutoland or liberia or libya or libia or jamahiriya or jamahiryia or madagascar or "malagasy republic" or malawi or nyasaland or mali or mauritania or mauritius or morocco or ifni or mozambique or "portuguese east africa" or mocambique or namibia or niger or nigeria or principe or "sao tome" or reunion or rwanda or ruanda or senegal or seychelles or "sierra leone" or somalia or "south africa" or "st helena" or sudan or tanzania or tanganyika or togo or "togolese republic" or tunisia or tunisie or uganda or "western sahara" or zambia or zimbabwe or "indian ocean islands" or sahara or magreb or magrib) not ("guinea pig" or "guinea pigs" or "aspergillus niger")) OR<br>AB ((africa* or algeria or algerie or angola or benin or dahomey or botswana or bechuanaland or "burkina faso" or "burkina fasso" or "upper volta" or "haute volta" or burundi or urundi or cameroon or cameroun or "canary islands" or "cape verde" or cabo verde or "ubangi shari" or chad or tchad or comoros or "comoro islands" or comores or mayotte or congo or zaire or djibouti or "french somaliland" or egypt or "united arab republic" or eritrea or eswatini or swaziland or ethiopia or gabon or "gabonese republic" or gambia or ghana or "gold coast" or guinea or "ivory coast" or "cote d#ivoire" or "cote d' ivoire" or kenya or lesotho or basutoland or liberia or libya or libia or jamahiriya or jamahiryia or madagascar or "malagasy republic" or malawi or nyasaland or mali or mauritania or mauritius or morocco or ifni or mozambique or "portuguese east africa" or mocambique or namibia or niger or nigeria or principe or "sao tome" or reunion or rwanda or ruanda or senegal or seychelles or "sierra leone" or somalia or "south africa" or "st helena" or sudan or tanzania or tanganyika or togo or "togolese republic" or tunisia or tunisie or uganda or "western sahara" or zambia or zimbabwe or "indian ocean islands" or sahara or magreb or magrib) not ("guinea pig" or "guinea pigs" or "aspergillus niger")) OR<br>SU ((africa* or algeria or algerie or angola or benin or dahomey or botswana or bechuanaland or "burkina faso" or "burkina fasso" or "upper volta" or "haute volta" or burundi or urundi or cameroon or cameroun or "canary islands" or "cape verde" or cabo verde or "ubangi shari" or chad or tchad or comoros or "comoro islands" or comores or mayotte or congo or zaire or djibouti or "french somaliland" or egypt or "united arab republic" or eritrea or eswatini or swaziland or ethiopia or gabon or "gabonese republic" or gambia or ghana or "gold coast" or guinea or "ivory coast" or "cote d#ivoire" or "cote d' ivoire" or kenya or lesotho or basutoland or liberia or libya or libia or jamahiriya or jamahiryia or madagascar or "malagasy republic" or malawi or nyasaland or mali or mauritania or mauritius or morocco or ifni or mozambique or "portuguese east africa" or mocambique or namibia or niger or nigeria or principe or "sao tome" or reunion or rwanda or ruanda or senegal or seychelles or "sierra leone" or somalia or "south africa" or "st helena" or sudan or tanzania or tanganyika or togo or "togolese republic" or tunisia or tunisie or uganda or "western sahara" or zambia or zimbabwe or "indian ocean islands" or sahara or magreb or magrib) not ("guinea pig" or "guinea pigs" or "aspergillus niger")) OR<br>AF ((africa* or algeria or algerie or angola or benin or dahomey or botswana or bechuanaland or "burkina faso" or "burkina fasso" or "upper volta" or "haute volta" or burundi or urundi or cameroon or cameroun or "canary islands" or "cape verde" or cabo verde or "ubangi shari" or chad or tchad or comoros or "comoro islands" or comores or mayotte or congo or zaire or djibouti or "french somaliland" or egypt or "united arab republic" or eritrea or eswatini or swaziland or ethiopia or gabon or "gabonese republic" or gambia or ghana or "gold coast" or guinea or "ivory coast" or "cote d#ivoire" or "cote d' ivoire" or kenya or lesotho or basutoland or liberia or libya or libia or jamahiriya or jamahiryia or madagascar or "malagasy republic" or malawi or nyasaland or mali or mauritania or mauritius or morocco or ifni or mozambique or "portuguese east africa" or mocambique or	S8	210,457

For peer review only - http://bmjopen.bmj.com/site/about/guidelines.xhtml

		namibia or niger or nigeria or principe or "sao tome" or reunion or rwanda or ruanda or senegal or seychelles or "sierra leone" or somalia or "south africa" or "st helena" or sudan or tanzania or tanganyika or togo or "togolese republic" or tunisia or tunisie or uganda or "western sahara" or zambia or zimbabwe or "indian ocean islands" or sahara or magreb or magrib) not ("guinea pig" or "guinea pigs" or "aspergillus niger"))		
	**All**	S7 or S8	S9	210,575
**Total result**		**S3 AND S6 AND S9**	**S10**	**899**

For peer review only - http://bmjopen.bmj.com/site/about/guidelines.xhtml

PsycINFO via Ovid (searched on August 29, 2022; Period: 1806 - August Week 3, 2022)

Concept		Keyword	umber	Result
**Equity**	**Controlled Vocabulary**	exp "gender identity"/ or exp "sexual minority groups"/ or exp disabilities/ or exp geriatrics/ or equity/ or "healthcare disparities"/ or "health care access"/ or "human sex differences"/ or "human females"/ or "human males"/ or neighborhoods/ or ghettoes/ or poverty areas/ or "rural environments"/ or "urban environments"/ or "minority groups"/ or "racial and ethnic differences"/ or "sociocultural factors"/ or "cross cultural differences"/ or "cultural identity"/ or "ethnic identity"/ or religion/ or "religious groups"/ or "social discrimination"/ or "socioeconomic factors"/ or "socioeconomic status"/ or "economic inequality"/ or "susceptibility (disorders)"/ or "at risk populations"/ or "social class"/ or disadvantaged/ or "educational attainment level"/ or unemployment/	1	619,023
	**Free Text**	(equit* or inequit* or inequalit* or disparit* or equalit* or deprivation or gini or "concentration index" or ((health* or medical) adj3 (availabl* or access*)) or (gender adj (difference? or identit* or role?)) or (sex adj (disparit* or difference? or role?)) or ((wom#n* or m#n*) adj role?) or "sexual minorit*" or "gender minorit*" or LGBT* or 2SLGBT* or urban or rural or ethnic* or religi* or ((social* or socio-economic or socioeconomic or economic or structural or material) adj3 (advantage* or disadvantage* or exclude* or exclusion or include* or inclusion or status or position or gradient* or hierarch* or class* or determinant*)) or (health adj3 (gap* or gradient* or hierarch*)) or SES or SEP or sociodemographic* or socio-demographic* or income or wealth* or poverty or "educational status" or "educational level?" or "level? of education" or "educational attainment" or ((well or better or higher or worse or less) adj (educated)) or unemploy* or "home owner*" or tenure or affluen* or "well off" or "better off" or "worse off").ti,ab,id.	2	698,649
	**All**	1 or 2	3	1,036,490
**Universal health coverage**	**Free Text**	(UHC or (universal adj2 (coverage or health* or care or access))).ti,ab,id.	4	1,840
**Africa**	**Free Text**	((africa* or algeria or algerie or angola or benin or dahomey or botswana or bechuanaland or "burkina faso" or "burkina fasso" or "upper volta" or "haute volta" or burundi or urundi or cameroon or cameroun or "canary islands" or "cape verde" or cabo verde or "ubangi shari" or chad or tchad or comoros or "comoro islands" or comores or mayotte or congo or zaire or djibouti or "french somaliland" or egypt or "united arab republic" or eritrea or eswatini or swaziland or ethiopia or gabon or "gabonese republic" or gambia or ghana or "gold coast" or guinea or "ivory coast" or "cote d?ivoire" or "cote d' ivoire" or kenya or lesotho or basutoland or liberia or libya or libia or jamahiriya or jamahiryia or madagascar or "malagasy republic" or malawi or nyasaland or mali or mauritania or mauritius or morocco or ifni or mozambique or "portuguese east africa" or mocambique or namibia or niger or nigeria or principe or "sao tome" or reunion or rwanda or ruanda or senegal or seychelles or "sierra leone" or somalia or "south africa" or "st helena" or sudan or tanzania or tanganyika or togo or "togolese republic" or tunisia or tunisie or uganda or "western sahara" or zambia or zimbabwe or "indian ocean islands" or sahara or magreb or magrib) not ("guinea pig" or "guinea pigs" or "aspergillus niger")).ti,ab,id,in	5	141,449
**Total result**		**3 AND 4 AND 5**	**6**	**168**

For peer review only - http://bmjopen.bmj.com/site/about/guidelines.xhtml

Cochrane Library (searched on August 29, 2022)

Concept		Keyword	umber	Result
**Equity**	**Controlled Vocabulary**	[mh "gender identity"] or [mh "sexual and gender minorities"] or [mh "disabled persons"] or [mh "geriatrics"] or [mh ^"health equity"] or [mh ^"health inequities"] or [mh ^"healthcare disparities"] or [mh ^"health status disparities"] or [mh ^"health services accessibility"] or [mh ^"sex factors"] or [mh ^"women"] or [mh ^"men"] or [mh ^"residence characteristics"] or [mh ^"urban population"] or [mh ^"rural population"] or [mh ^"cultural characteristics"] or [mh ^"cultural diversity"] or [mh ^"religion"] or [mh ^"social discrimination"] or [mh ^"socioeconomic factors"] or [mh ^"vulnerable populations"] or [mh ^"social class"] or [mh ^"minority groups"] or [mh ^"ethnic and racial minorities"] or [mh ^"educational status"] or [mh ^"unemployment"]	#1	18,168
	**Free Text**	(equit* or inequit* or inequalit* or disparit* or equalit* or deprivation or gini or "concentration index" or ((health* or medical) NEAR/2 (availabl* or access*)) or (gender NEXT (difference? or identit* or role?)) or (sex NEXT (disparit* or difference? or role?)) or ((wom?n* or m?n*) NEXT role?) or "sexual minorit*" or "gender minorit*" or LGBT* or 2SLGBT* or urban or rural or ethnic* or religi* or ((social* or socio-economic or socioeconomic or economic or structural or material) NEAR/2 (advantage* or disadvantage* or exclude* or exclusion or include* or inclusion or status or position or gradient* or hierarch* or class* or determinant*)) or (health NEAR/2 (gap* or gradient* or hierarch*)) or SES or SEP or sociodemographic* or socio-demographic* or income or wealth* or poverty or "educational status" or "educational level?" or "level? of education" or "educational attainment" or ((well or better or higher or worse or less) NEXT (educated)) or unemploy* or "home owner*" or tenure or affluen* or "well off" or "better off" or "worse off"):ti,ab,kw	#2	79,541
	**All**	#1 or #2	#3	87,932
**Universal health coverage**	**Controlled Vocabulary**	[mh ^"universal health care"]	#4	0
	**Free Text**	(UHC or (universal NEAR/1 (coverage or health* or care or access))):ti,ab,kw	#5	306
	**All**	#4 or #5	#6	306
**Africa**	**Controlled Vocabulary**	[mh "africa"] not ("guinea pig" or "guinea pigs" or "aspergillus niger"):ti,ab,kw	#7	8,273
	**Free Text**	((africa* or algeria or algerie or angola or benin or dahomey or botswana or bechuanaland or "burkina faso" or "burkina fasso" or "upper volta" or "haute volta" or burundi or urundi or cameroon or cameroun or "canary islands" or "cape verde" or cabo verde or "ubangi shari" or chad or tchad or comoros or "comoro islands" or comores or mayotte or congo or zaire or djibouti or "french somaliland" or egypt or "united arab republic" or eritrea or eswatini or swaziland or ethiopia or gabon or "gabonese republic" or gambia or ghana or "gold coast" or guinea or "ivory coast" or "cote d?ivoire" or "cote d' ivoire" or kenya or lesotho or basutoland or liberia or libya or libia or jamahiriya or jamahiryia or madagascar or "malagasy republic" or malawi or nyasaland or mali or mauritania or mauritius or morocco or ifni or mozambique or "portuguese east africa" or mocambique or namibia or niger or nigeria or principe or "sao tome" or reunion or rwanda or ruanda or senegal or seychelles or "sierra leone" or somalia or "south africa" or "st helena" or sudan or tanzania or tanganyika or togo or "togolese republic" or tunisia or tunisie or uganda or "western sahara" or zambia or zimbabwe or "indian ocean islands" or sahara or magreb or magrib) not ("guinea pig" or "guinea pigs" or "aspergillus niger")):ti,ab,kw	#8	34,555
	**All**	#7 or #8	#9	34,555
**Total result**		**#3 AND #6 AND #9**	**#10**	**56**

For peer review only - http://bmjopen.bmj.com/site/about/guidelines.xhtml

BMJ Open

Mapping health service coverage inequalities in Africa: a scoping review protocol

Journal:	*BMJ Open*
Manuscript ID	bmjopen-2022-068903.R1
Article Type:	Protocol
Date Submitted by the Author:	01-Mar-2023
Complete List of Authors:	Karamagi, Humphrey Cyprian ; World Health Organization Regional Office for Africa,<br>Ben Charif, Ali; Université Laval,<br>Afriyie, Doris; Swiss Tropical and Public Health Institute, Epidemiology and Public Health; University of Basel,<br>SY, Sokona; World Health Organization Regional Office for Africa, Data, Analytics and Knowledge Management<br>kipruto, Hillary; World Health Organization Regional Office for Africa, Inter-Country Support Team for Eastern & Southern Africa<br>Oyelade, Taiwo; World Health Organization Regional Office for Africa<br>Droti, Benson; World Health Organization Regional Office for Africa
**Primary Subject Heading**:	Qualitative research
Secondary Subject Heading:	Health services research
Keywords:	Protocols & guidelines < HEALTH SERVICES ADMINISTRATION & MANAGEMENT, PUBLIC HEALTH, Health Equity

SCHOLARONE™
Manuscripts

Mapping health service coverage inequalities in Africa: a scoping review protocol

Humphrey Cyprian KARAMAGI[1]*, Ali BEN CHARIF[2], Doris OSEI AFRIYIE[3], Sokona SY[1], Hillary Kipchumba KIPRUTO[4], Taiwo OYELADE[5], Benson DROTI[4]

Institutional addresses

[1]Data Analytics and Knowledge Management, World Health Organization (WHO) Regional Office for Africa, Brazzaville, Republic of Congo; [2]CubecXpert, Quebec City, QC, Canada; [3]Department of Epidemiology and Public Health, Swiss Tropical and Public Health Institute, Basel, Switzerland; [4]Health Information Systems, World Health Organization (WHO) Regional Office for Africa, InterCountry Support team for Eastern and Southern Africa Harare, Zimbabwe;[5]World Health Organization (WHO) Regional Office for Africa, Brazzaville, Republic of Congo;

Email addresses for all authors

HCK, karamagih@gmail.com
ABC, ali.bencharif@cubecxpert.ca
DOA, doriso28@gmail.com
SS, sys@who.int
HKK, kiprutohi@who.int
TO, oyeladet@who.int
BD, drotib@who.int

***Corresponding author:** Dr. Humphrey Cyprian KARAMAGI
World Health Organization (WHO) Regional Office for Africa, Brazzaville, Republic of Congo | karamagih@gmail.com

Word Count: 2,392/4,000 words | **Abstract:** 300/300 words | **Figures:** 0 | **Tables:** 1 | **References:** 37.

For peer review only

For peer review only - http://bmjopen.bmj.com/site/about/guidelines.xhtml

Abstract

Introduction: Addressing inequities in health service coverage is a global priority, especially with the resurgence of interest in universal health coverage. However, in Africa, which has the lowest health service coverage index, there is limited information on the progress of countries in addressing inequalities related to health services. Thus, we seek to map the evidence on inequalities in health service coverage in Africa.

Methods and analysis: We will conduct a scoping review following the Joanna Briggs Institute (JBI) Manual for Evidence Synthesis. We preregistered this protocol with the Open Science Framework on July 26, 2022 (https://osf.io/zd5bt). We will consider any empirical research that assesses inequalities in relation to services for reproductive, maternal, newborn and child health (e.g., family planning), infectious diseases (e.g., tuberculosis treatment), and non-communicable diseases (e.g., cervical cancer screening) in Africa. We will search MEDLINE, Embase, Web of Science, CINAHL, PsycINFO, and Cochrane Library from their inception onwards. We will also hand-search Google and Global Index Medicus, and screen reference lists of relevant studies. We will evaluate studies for eligibility and extract data from included studies using pre-piloted and standardized forms. We will further extract a core set of health service coverage indicators, which are disaggregated by PROGRESS-Plus equity stratifiers. We will summarize data using a narrative approach involving thematic syntheses and descriptive statistics. We will report our findings according to the Preferred Reporting Items for Systematic Reviews and Meta-Analysis (PRISMA) extension for Scoping Reviews checklist.

Ethics and dissemination: Ethical approval is not required as primary data will not be collected. This work will contribute to identifying knowledge gaps in the evidence of inequalities in health service coverage in Africa, and propose strategies that could help overcome current challenges. We will disseminate our findings to knowledge users through a publication in a peer-reviewed journal and organization of workshops.

Keywords: Inequality, equality, equity, inequity, disparity, deprivation, PROGRESS-Plus, universal health coverage, Africa, scoping review.

For peer review only - http://bmjopen.bmj.com/site/about/guidelines.xhtml

Strengths and limitations of this study

1. This scoping review will follow the Joanna Briggs Institute (JBI) Manual for Evidence Synthesis.
2. We will conduct a comprehensive literature on multiple electronic databases on inequalities and service coverage in Africa.
3. We will use PROGRESS-Plus to guide our search strategy and conceptualization of inequality.
4. We will adapt the service coverage indicators used in tracking universal health coverage by the World Health Organization and World Bank.
5. We foresee extensive data given the broad indicators for service coverage.

For peer review only

For peer review only - http://bmjopen.bmj.com/site/about/guidelines.xhtml

Introduction

The attainment of good health and well-being has been prioritized as a common goal by African countries, as outlined in the third Sustainable Development Goal (SDG 3) established by the United Nations in 2015 [1]. Within the World Health Organization (WHO) Regional Office for Africa, countries have recognized attainment of universal health coverage (UHC) as a critical outcome necessary to attain this goal along with good health security and coverage of health determinants [2,3]. One of the main goals of universal health coverage is to ensure that all people receive the health services they need, including promotive, preventative, curative, rehabilitative, and palliative care which are of sufficient quality [4]. At the core of universal health coverage goals is a commitment to health equity.

WHO defines health equity as "the absence of unfair and avoidable or remediable differences in health among population groups defined socially, economically, demographically or geographically" [5]. In principle, health inequities are systematic differences that are socially produced, and put groups disadvantaged already at further disadvantage related to their health [6]. A key step towards addressing and assessing health equities is monitoring health inequalities-health differences between population subgroups [7].

The roots of inequalities in health can be complex and influenced by a myriad of social conditions. In 2005, the WHO commission on social determinants of health emphasized the role of structural mechanisms, which create stratification and social class divisions

For peer review only - http://bmjopen.bmj.com/site/about/guidelines.xhtml

that shape the health opportunities of various social groups based on their level of power, prestige and access to resources [5]. The commission identified six important structural stratifiers: 1) income, 2) education, 3) occupation, 4) social class, 5) gender, and 6) race or ethnicity. Additionally, other studies in sub-Saharan Africa have also recognized the need to include historical and cultural context, which underlies causal factors for the social determinants of health in the region [8,9].

Few reviews have specifically examined inequalities within the context of universal health coverage. The limited reviews that have been conducted have mainly focused on specific services such as Reproductive, Maternal, Newborn and Child health (RMNCH) services [10,11]. Additionally, others have assessed inequalities using selected stratifiers, such as socioeconomic status and age [12,13]. In this review, we seek to consolidate the evidence on service coverage inequalities in Africa using a comprehensive set of stratifiers to assess these inequalities. The specific objectives of the review are to: 1) Outline the methodological approaches used in assessing health inequalities in relation to service coverage; 2) Characterize the current evidence on service coverage inequalities; 3) Identify knowledge gaps in the existing evidence on service coverage inequalities); 4) Document effective strategies being used to tackling the different drivers of inequalities in service coverage; and 5) Identify challenges related to addressing health equalities in Africa.

Methods and analysis

Design

For peer review only - http://bmjopen.bmj.com/site/about/guidelines.xhtml

We will conduct a scoping review following the methodology recommended in the Joanna Briggs Institute (JBI) Manual for Evidence Synthesis [14]. This methodology is based on the Arksey and O'Malley framework [15] and an enhanced version developed by Levac and colleagues [16] for conducting scoping reviews [17]. A scoping was selected as we aim to outline the evidence in the area of interest and identify knowledge gaps. This protocol has been registered with the Open Science Framework (OSF) on July 26, 2022 (identifier: https://osf.io/zd5bt). We searched our electronic databases on August 29, 2022, and plan to complete this review by June 26, 2023. We will report this review according to the Preferred Reporting Items for Systematic Reviews and Meta-Analysis (PRISMA) extension for Scoping Reviews (PRISMA-ScR) checklist [18]. In this protocol, we use the standard PRISMA definitions for a report [19]. We report the content for this scoping review protocol using the PRISMA Protocol checklist (**Appendix 1**) [20].

Eligibility criteria

Following the Joanna Briggs Institute Manual for Evidence Synthesis [14], we will use the following eligibility criteria:

- **Participants:** We will consider studies involving individuals, communities, or organizations involved in the receipt of health services within a health system context in Africa. No restrictions based on socio-demographic factors (e.g., sex, age, and ethnicity) or health conditions (e.g.; comorbidities) will be applied. We will consider any countries or geographic regions in the African continent such as the 47 Member States of the WHO Regional Office for Africa, the Maghreb, and all other African regions. Global studies which include both an African region and

For peer review only - http://bmjopen.bmj.com/site/about/guidelines.xhtml

For peer review only

other regions from other continents (e.g., Europe, Asia, or America) will be considered. We will exclude studies involving only regions or countries outside the African geographic region.

- **Concept:** We will consider studies assessing inequalities or differences in health service coverage between subgroups. Health service coverage refers to the access to or use of health services (i.e., equal service for equal need) [21,22]. To identify stratifiers, we will adopt the Cochrane and Campbell Equity Methods group framework of PROGRESS-Plus (place of residence, race/ethnicity/culture, occupation, gender, religion, education, socioeconomic status, and social capital). This framework includes social factors that can influence health disparities and the "Plus" includes other factors in which health inequalities may exist such as age, disability, and sexual orientation. We will exclude studies that did not examine inequalities in health service coverage, such as studies on health financing or financial protection, an area overviewed in the literature. However, studies which assess financial hardship as a driver of inequalities of health service will be included.

Context: We will consider studies that assess the use of essential health services. We will adapt the WHO and World Bank indicators for health service coverage and will consider essential health services within three components: 1) Reproductive, maternal, newborn and child health (family planning, antenatal care, delivery care, postnatal care, child immunization, and health-seeking behaviour for pneumonia); 2) Infectious diseases (tuberculosis treatment, human immunodeficiency virus (HIV) therapy, use of insecticide-treated bed nets for

For peer review only - http://bmjopen.bmj.com/site/about/guidelines.xhtml

160 malaria prevention, adequate sanitation, and neglected tropical diseases treatment); and 3) Non-communicable diseases (prevention and treatment of raised blood pressure, prevention and treatment of raised blood glucose, cervical cancer screening, and tobacco (non-)smoking) (Appendix 2). We will also consider any index or sub-index assessing health service coverage of these components (e.g., sub index on infectious disease services). However, we will exclude indicators or indices related to basic hospital access, health worker density, access to essential medicines, or health security.

- **Types of sources:** We will consider empirical studies using qualitative, quantitative, or mixed methods designs. This includes knowledge syntheses (e.g., systematic reviews), experimental, quasi-experimental, and observational designs. No restrictions will be placed on the language of publication or publication status. However, we will exclude studies published before 2005, because that is the year the term "Universal Coverage" was mentioned in a World Health Assembly resolution [23]. Additionally, we will also exclude any retracted publications, conference abstracts, study protocols, and editorial materials (e.g., editorials, commentaries, and letters).

In essence, we will consider any empirical research that utilizes any study designs and measures inequalities in relation to services for services for reproductive, maternal, newborn and child health (e.g., family planning), infectious diseases (e.g., tuberculosis treatment), and non-communicable diseases (e.g., cervical cancer screening) in Africa (Table1).

For peer review only - http://bmjopen.bmj.com/site/about/guidelines.xhtml

Table 1. Criteria for considering studies for this review

Criteria	Inclusion	Exclusion
**Participants**	Individuals, communities, or organizations involved in the receipt of health care services within a health system context in Africa	⊗ Any countries or regions outside the African geographic region
**Concept**	Studies focusing on one of the following PROGRESS-Plus* equity stratifier:<br>▯ Place of residence (e.g., rural, urban)<br>▯ Race, ethnicity, or culture<br>▯ Occupation<br>▯ Gender or sex<br>▯ Religion<br>▯ Education<br>▯ Socioeconomic status<br>▯ Social capital or resources<br>▯ Any other factors in which health inequalities may exist (e.g., age, disability, and sexual orientation).	⊗ Studies that did not include a PROGRESS-Plus* equity stratifier<br>⊗ Studies that did not examine inequalities in health service coverage<br>⊗ Studies focusing on inequality in health financing or financial protection
**Context**	Studies monitoring at least one indicator related to the following essential health services (Appendix 2):<br>▯ Family planning<br>▯ Antenatal, delivery, and postnatal care<br>▯ Child immunization<br>▯ Health-seeking behaviour for pneumonia<br>▯ Tuberculosis treatment<br>▯ Human immunodeficiency virus (HIV) therapy<br>▯ Use of insecticide-treated bed nets for malaria prevention<br>▯ Adequate sanitation<br>▯ Neglected tropical diseases treatment and care<br>▯ Prevention and treatment of raised blood pressure<br>▯ Prevention and treatment of raised blood glucose<br>▯ Cervical cancer screening<br>▯ Tobacco (non-)smoking	⊗ Studies that did not include any of those essential health services<br>⊗ Studies focusing on basic hospital access<br>⊗ Studies focusing on health workforce<br>⊗ Studies focusing on access to essential medicines<br>⊗ Studies focusing on health security
**Type of sources**	Empirical studies using a quantitative, qualitative, or mixed methods design and published from 2005:<br>▯ Original studies from 2005 onwards<br>▯ Conference articles from 2005 onwards<br>▯ Knowledge syntheses from 2005 onwards	⊗ Protocols<br>⊗ Conference abstracts<br>⊗ Retracted publications<br>⊗ Records published before 2005<br>⊗ Editorial materials (e.g., commentary, letter, editorials)

Abbreviation: PROGRESS: place of residence, race/ethnicity/culture, occupation, gender, religion, education, socioeconomic status, and social capital.
Notes: *The "Plus" includes other factors in which health inequalities may exist such as age, disability, and sexual orientation.

Literature search

For peer review only

For peer review only - http://bmjopen.bmj.com/site/about/guidelines.xhtml

We will perform a comprehensive search to identify records through electronic databases and other relevant sources. No restrictions will be placed on date of publication, language, place of publication, or type of reports in our search strategy.

We will search MEDLINE, Embase, Web of Science, CINAHL, PsycINFO, and the Cochrane Library from their dates of inception onwards. We will perform the preliminary search strategy in Ovid MEDLINE following appropriate design principles [24]. An information specialist and our core team of international experts in health equity, universal health coverage, health information systems, or knowledge syntheses from Africa will review this preliminary search strategy. The search terms will be adapted to the above-mentioned databases. The search terms will be based on previous works to reflect three concepts: 1) inequality, 2) health service coverage, and 3) African regions **(Appendix 3)**. For inequality, we will adapt a validated search filter [25], by including adding other terms that are suitable for the African context (e.g., rural and religion) [26–28]. For service coverage, we will use terms related to universal health coverage that were previously identified with an exploratory search in Google, Google Scholar, and Abstract reviews [29]. For African geographic regions, we will adapt a geographic African filter validated by the South African Cochrane Centre [30], by correcting the name “Mayotte” and including alternative missing African country names (e.g., “Ruanda”, “Comoros”, and “Cabo Verde”). We will use the list of African region names used to develop the low- and middle-income countries geographic search filter by the Cochrane Effective Practice and Organisation of Care (EPOC), in collaboration with the WHO and Campbell Collaboration [31,32].

For peer review only - http://bmjopen.bmj.com/site/about/guidelines.xhtml

In addition to electronic databases, we will also identify relevant records through screening reference lists of relevant reports and hand searching on Google and WHO Global Index Medicus. From the results of the two websites, we will screen at least the first 30 results for each search. Previous experiences show that results beyond the first 30 results are often duplicates and unlikely to be relevant [33,34].

Selection of sources of evidence

Following of the search, we will collate and upload all the records all the records into EndNote 20 (Clarivate Analytics, PA, USA), and remove duplicates. Screening forms, standardized in Google Sheets will be prepared based on eligibility criteria refined by the entire review team to ensure accurate selection of eligible records. As suggested by Joanna Briggs Institute, we will select a random sample of 25 records for the pilot test and only start screening when agreement of 75% or greater is achieved. We will calculate the inter-reviewer agreement using the weighted Cohen's kappa [35]. One reviewer will screen all remaining records and identify potentially relevant reports that meet the eligibility criteria. Each record or report will be screened by one using the standardized forms and checked by another. We will document a reason for excluding any ineligible report. Any discrepancies will be resolved through consensus or with the assistance of a third reviewer.

Data charting process

We will develop a form in Google sheets in consultation with the core team to guide the extraction of variables. Two reviewers will independently perform a pilot test of the form to ensure it captures relevant data. We will extract the following information: study characteristics (e.g., title, authors, year of publication, design, target participants,

For peer review only - http://bmjopen.bmj.com/site/about/guidelines.xhtml

country); inequality dimensions (e.g., PROGRESS-Plus elements); methodological approaches used to measure inequalities (e.g., indices); and health service coverage indicators (e.g., skilled birth attendance, complete antenatal care visits). Full charting will be completed by one reviewer and checked by another. Any discrepancies between reviewers will be resolved by discussion or with the assistance of a third reviewer.

Critical appraisal

Due to the nature of our research question, we will not perform an appraisal for risk of bias or conduct quality assessment. This is consistent with the Joanna Briggs Institute (JBI) Manual for Evidence Synthesis [14]. A critical appraisal is generally not recommended in scoping reviews because the aim is to map the available evidence rather than provide a synthesized and clinically meaningful answer to our research question [36].

Synthesis of results

We will employ both qualitative and quantitative methods to analyze the data generated. A descriptive summary of the characteristics of included studies will be presented [16]. We will map studies according to the appropriate health service indicators and PROGRESS-Plus elements. Additionally, we will undertake a qualitative synthesis to identify common themes among included studies on the evidence in the findings and probable explanations for service coverage inequalities in the discussion sections [16]. We will use the PRISMA 2020 flowchart to describe the process of report selection [19].

Patient and Public Involvement

For peer review only - http://bmjopen.bmj.com/site/about/guidelines.xhtml

Patients, and the public will not be involved in the design, conduct, or parting of this scoping review. However, we will adopt an integrated knowledge translation approach, where policy makers, clinicians, researchers, and trainees are equal members. This will ensure that the research is relevant and useful to knowledge users, increasing the likelihood of uptake [37]. This will involve female and male international African experts. They will be engaging virtually once a week to discuss status and progress that will be transparently available to all members using google drive. Multidisciplinary consultations will also be conducted with policy-level experts who will be purposively selected based on the topic for analysis, to enrich the interpretation of findings.

Discussion

This scoping review seeks to map the evidence on the state of inequality in the progress towards universal health service coverage and its constituent components in Africa. It will fill an important gap by providing a comprehensive body of evidence that exists on the progress towards equity in universal health coverage across Africa. First, it will be possible to map the evidence on inequalities in coverage for the broad range of essential services across several dimensions of inequalities. Second, it will be able to highlight the barriers and opportunities for effectively addressing the drivers of health inequalities in Africa. Third, it will be possible to demonstrate the appropriate methodological approaches for measuring health inequalities in the African context. Lastly, it will highlight which essential services and its relevant dimensions of inequalities that have been under-researched in the literature, and may need future investigations. Thus, this review will make a critical contribution to monitoring inequalities in health service

For peer review only - http://bmjopen.bmj.com/site/about/guidelines.xhtml

coverage promoting learning and building the evidence for investments in effective strategies to reduce health disparities in Africa.

Ethics and dissemination

This scoping review will involve neither human participants nor unpublished secondary data. As such, formal ethical approval from a research ethics committee is not required. We will disseminate our results through publications in peer-reviewed journals, and a technical report for the WHO Regional Office for Africa. We will also share our reports using free public repositories such as Open Science Framework and ResearchGate.

For peer review only - http://bmjopen.bmj.com/site/about/guidelines.xhtml

For peer review only

List of abbreviations

PRISMA: Preferred Reporting Items for Systematic Reviews and Meta-Analysis;
PRISMA-ScR: PRISMA extension for Scoping Reviews;
PROGRESS: Place of residence, Race/ethnicity/culture, Occupation, Gender, Religion, Education, Socioeconomic status, and Social capital
WHO: World Health Organization;

Declarations

Ethics approval and consent to participate
Not applicable.

Consent for publication
Not applicable.

Availability of data and materials
Please send all requests for study data or materials to Dr. Humphrey Cyprian KARAMAGI (karamagih@gmail.com).

Competing interests
Authors declare that they have no competing interests.

Funding
No direct funding was provided.

Authors' contributions
HCK, SS, HKK, TO, and BD conceptualized the study. HCK, ABC, DOA, SS, and HKK participated in the design of this protocol. ABC drafted the search strategy and all authors revised it. ABC and DOA drafted this protocol. All authors revised the manuscript critically for important intellectual content, gave final approval of the version to be published and agreed to be accountable for all aspects of the knowledge synthesis.

Acknowledgements
We wish to acknowledge the World Health Organization (WHO) Regional Office for Africa for their assistance with various aspects of this work.

For peer review only - http://bmjopen.bmj.com/site/about/guidelines.xhtml

References

1 General Assembly. Resolution adopted by the General Assembly on 11 September 2015. New York: : United Nations 2015.

2 Karamagi HC, Tumusiime P, Titi-Ofei R, *et al.* Towards universal health coverage in the WHO African Region: assessing health system functionality, incorporating lessons from COVID-19. *BMJ Global Health* 2021;**6**:e004618. doi:10.1136/bmjgh-2020-004618

3 Karamagi HC, Ben Charif A, Kidane SN, *et al.* Investments for effective functionality of health systems towards Universal Health Coverage in Africa: A scoping review. *PLOS Global Public Health* 2022;**2**:e0001076. doi:10.1371/journal.pgph.0001076

4 World Health Organization, World Bank. Tracking Universal Health Coverage: 2021 Global Monitoring Report. Washington, DC: : World Bank 2021. https://openknowledge.worldbank.org/handle/10986/36724 (accessed 5 Jul 2022).

5 World Health Organization. A conceptual framework for action on the social determinants of health. World Health Organization 2010. https://apps.who.int/iris/handle/10665/44489 (accessed 5 Jul 2022).

6 Braveman P, Gruskin S. Defining equity in health. *J Epidemiol Community Health* 2003;**57**:254–8. doi:10.1136/jech.57.4.254

7 Hanson K, Brikci N, Erlangga D, *et al.* The Lancet Global Health Commission on financing primary health care: putting people at the centre. *The Lancet Global Health* 2022;**10**:e715–72. doi:10.1016/S2214-109X(22)00005-5

8 Eshetu E, Woldesenbet S. Are there particular social determinants of health for the world's poorest countries? *Afr Health Sci* 2011;**11**:108–15.

9 Ichoku HE, Mooney G, Ataguba JE-O. Africanizing the social determinants of health: embedded structural inequalities and current health outcomes in sub-Saharan Africa. *Int J Health Serv* 2013;**43**:745–59. doi:10.2190/HS.43.4.i

10 Çalışkan Z, Kılıç D, Öztürk S, *et al.* Equity in maternal health care service utilization: a systematic review for developing countries. *Int J Public Health* 2015;**60**:815–25. doi:10.1007/s00038-015-0711-x

11 Målqvist M, Yuan B, Trygg N, *et al.* Targeted Interventions for Improved Equity in Maternal and Child Health in Low- and Middle-Income Settings: A Systematic Review and Meta-Analysis. *PLOS ONE* 2013;**8**:e66453. doi:10.1371/journal.pone.0066453

For peer review only - http://bmjopen.bmj.com/site/about/guidelines.xhtml

12 Gao Q, Prina AM, Ma Y, *et al.* Inequalities in Older age and Primary Health Care Utilization in Low- and Middle-Income Countries: A Systematic Review. *Int J Health Serv* 2022;**52**:99–114. doi:10.1177/00207314211041234

13 Hanratty B, Zhang T, Whitehead M. How close have universal health systems come to achieving equity in use of curative services? A systematic review. *Int J Health Serv* 2007;**37**:89–109. doi:10.2190/TTX2-3572-UL81-62W7

14 Peters M, Godfrey C, McInerney P, *et al.* Chapter 11: Scoping Reviews. In: Aromataris E, Munn Z, eds. *JBI Manual for Evidence Synthesis*. JBI 2020. doi:10.46658/JBIMES-20-12

15 Arksey H, O'Malley L. Scoping studies: towards a methodological framework. *International Journal of Social Research Methodology* 2005;**8**:19–32. doi:10.1080/1364557032000119616

16 Levac D, Colquhoun H, O'Brien KK. Scoping studies: advancing the methodology. *Implementation Science* 2010;**5**:69. doi:10.1186/1748-5908-5-69

17 Munn Z, Pollock D, Khalil H, *et al.* What are scoping reviews? Providing a formal definition of scoping reviews as a type of evidence synthesis. *JBI Evidence Synthesis* 2022;**20**:950–2. doi:10.11124/JBIES-21-00483

18 Tricco AC, Lillie E, Zarin W, *et al.* PRISMA Extension for Scoping Reviews (PRISMA-ScR): Checklist and Explanation. *Ann Intern Med* 2018;**169**:467–73. doi:10.7326/M18-0850

19 Page MJ, McKenzie JE, Bossuyt PM, *et al.* The PRISMA 2020 statement: an updated guideline for reporting systematic reviews. *BMJ* 2021;**372**:n71. doi:10.1136/bmj.n71

20 Moher D, Shamseer L, Clarke M, *et al.* Preferred reporting items for systematic review and meta-analysis protocols (PRISMA-P) 2015 statement. *Syst Rev* 2015;**4**:1. doi:10.1186/2046-4053-4-1

21 Cohen AB, Grogan CM, Horwitt JN. The Many Roads toward Achieving Health Equity. *Journal of Health Politics, Policy and Law* 2017;**42**:739–48. doi:10.1215/03616878-3940414

22 Paul E, Deville C, Bodson O, *et al.* How is equity approached in universal health coverage? An analysis of global and country policy documents in Benin and Senegal. *International Journal for Equity in Health* 2019;**18**:195. doi:10.1186/s12939-019-1089-9

23 World Health Assembly 58. Social health insurance: sustainable health financing, universal coverage and social health insurance: report by the Secretariat. World Health Organization 2005. https://apps.who.int/iris/handle/10665/20302 (accessed 3 Aug 2022).

For peer review only - http://bmjopen.bmj.com/site/about/guidelines.xhtml

24 MacFarlane A, Russell-Rose T, Shokraneh F. Search strategy formulation for systematic reviews: Issues, challenges and opportunities. *Intelligent Systems with Applications* 2022;**15**:200091. doi:10.1016/j.iswa.2022.200091

25 Hosking J, Macmillan A, Jones R, *et al.* Searching for health equity: validation of a search filter for ethnic and socioeconomic inequalities in transport. *Systematic Reviews* 2019;**8**:94. doi:10.1186/s13643-019-1009-5

26 Parker R, Wanner A, Foster M, *et al.* Design and validation of a search filter for LGBTQ+ populations. In: *25th Cochrane Colloquium*. Edinburgh, UK: 2018. 16–8.https://doi.org/10.1002/14651858.CD201801

27 Rizvi A, Lawson DO, Young T, *et al.* Guidance relevant to the reporting of health equity in observational research: a scoping review protocol. *BMJ Open* 2022;**12**:e056875. doi:10.1136/bmjopen-2021-056875

28 Prady SL, Uphoff EP, Power M, *et al.* Development and validation of a search filter to identify equity-focused studies: reducing the number needed to screen. *BMC Medical Research Methodology* 2018;**18**:106. doi:10.1186/s12874-018-0567-x

29 Rizvi SS, Douglas R, Williams OD, *et al.* The political economy of universal health coverage: a systematic narrative review. *Health Policy and Planning* 2020;**35**:364–72. doi:10.1093/heapol/czz171

30 Pienaar E, Grobler L, Busgeeth K, *et al.* Developing a geographic search filter to identify randomised controlled trials in Africa: finding the optimal balance between sensitivity and precision. *Health Info Libr J* 2011;**28**:210–5. doi:10.1111/j.1471-1842.2011.00936.x

31 Cochrane Effective Practice and Organisation of Care (EPOC). Low- and middle-income country (LMIC) filters. 2020.https://epoc.cochrane.org/lmic-filters (accessed 25 Nov 2021).

32 Sutton A, Campbell F. The ScHARR LMIC filter: Adapting a low- and middle-income countries geographic search filter to identify studies on preterm birth prevention and management. *Res Synth Methods* 2022;**13**:447–56. doi:10.1002/jrsm.1552

33 Hargrave DR, Hargrave UA, Bouffet E. Quality of health information on the Internet in pediatric neuro-oncology. *Neuro-oncol* 2006;**8**:175–82. doi:10.1215/15228517-2005-008

34 van der Marel S, Duijvestein M, Hardwick JC, *et al.* Quality of web-based information on inflammatory bowel diseases. *Inflamm Bowel Dis* 2009;**15**:1891–6. doi:10.1002/ibd.20976

For peer review only - http://bmjopen.bmj.com/site/about/guidelines.xhtml

35 Cohen J. Weighted kappa: Nominal scale agreement provision for scaled disagreement or partial credit. *Psychological Bulletin* 1968;**70**:213–20. doi:10.1037/h0026256

36 Peters MDJ, Marnie C, Tricco AC, *et al.* Updated methodological guidance for the conduct of scoping reviews. *JBI Evidence Synthesis* 2020;**18**:2119. doi:10.11124/JBIES-20-00167

37 Canadian Institutes of Health Research (CIHR). Guide to Knowledge Translation Planning at CIHR: Integrated and End-of-Grant Approaches. 2012.http://www.cihr-irsc.gc.ca/e/45321.html#a5 (accessed 28 Feb 2018).

For peer review only

For peer review only - http://bmjopen.bmj.com/site/about/guidelines.xhtml

Tables

Table 1. Criteria for considering studies for this review

Additional files

Appendix 1. The PRISMA-P 2015 Checklist

Appendix 2. Health Service Coverage Indicators

Appendix 3. Electronic Search strategies for Databases

For peer review only

For peer review only - http://bmjopen.bmj.com/site/about/guidelines.xhtml

PRISMA-P 2015 Checklist

This checklist has been adapted for use with protocol submissions to *Systematic Reviews* from Table 3 in Moher D et al: Preferred reporting items for systematic review and meta-analysis protocols (PRISMA-P) 2015 statement. *Systematic Reviews* 2015 **4**:1

Section/topic	#	Checklist item	Information reported		Line number(s)
			Yes	No	
**ADMINISTRATIVE INFORMATION**					
**Title**					
Identification	1a	Identify the report as a protocol of a systematic review	☒	☐	1
Update	1b	If the protocol is for an update of a previous systematic review, identify as such	☐	☐	Not applicable
**Registration**	2	If registered, provide the name of the registry (e.g., PROSPERO) and registration number in the Abstract	☒	☐	40
**Authors**					
Contact	3a	Provide name, institutional affiliation, and e-mail address of all protocol authors; provide physical mailing address of corresponding author	☒	☐	6-27
Contributions	3b	Describe contributions of protocol authors and identify the guarantor of the review	☒	☐	311-319
**Amendments**	4	If the protocol represents an amendment of a previously completed or published protocol, identify as such and list changes; otherwise, state plan for documenting important protocol amendments	☐	☐	Not applicable
**Support**					
Sources	5a	Indicate sources of financial or other support for the review	☒	☐	308-309
Sponsor	5b	Provide name for the review funder and/or sponsor	☒	☐	308-309
Role of sponsor/funder	5c	Describe roles of funder(s), sponsor(s), and/or institution(s), if any, in developing the protocol	☒	☐	308-309
**INTRODUCTION**					
**Rationale**	6	Describe the rationale for the review in the context of what is already known	☒	☐	71-105
**Objectives**	7	Provide an explicit statement of the question(s) the review will address with reference to participants, interventions, comparators, and outcomes (PICO)	☒	☐	105-111

For peer review only

BioMed Central
The Open Access Publisher

Section/topic	#	Checklist item	Information reported		Line number(s)
			Yes	No	
**METHODS**					
**Eligibility criteria**	8	Specify the study characteristics (e.g., PICO, study design, setting, time frame) and report characteristics (e.g., years considered, language, publication status) to be used as criteria for eligibility for the review	☒	☐	131-187
**Information sources**	9	Describe all intended information sources (e.g., electronic databases, contact with study authors, trial registers, or other grey literature sources) with planned dates of coverage	☒	☐	187-214
**Search strategy**	10	Present draft of search strategy to be used for at least one electronic database, including planned limits, such that it could be repeated	☒	☐	287-214
STUDY RECORDS					
Data management	11a	Describe the mechanism(s) that will be used to manage records and data throughout the review	☒	☐	113-227
Selection process	11b	State the process that will be used for selecting studies (e.g., two independent reviewers) through each phase of the review (i.e., screening, eligibility, and inclusion in meta-analysis)	☒	☐	215-227
Data collection process	11c	Describe planned method of extracting data from reports (e.g., piloting forms, done independently, in duplicate), any processes for obtaining and confirming data from investigators	☒	☐	228-237
**Data items**	12	List and define all variables for which data will be sought (e.g., PICO items, funding sources), any pre-planned data assumptions and simplifications	☒	☐	228-237
**Outcomes and prioritization**	13	List and define all outcomes for which data will be sought, including prioritization of main and additional outcomes, with rationale	☒	☐	228-237
**Risk of bias in individual studies**	14	Describe anticipated methods for assessing risk of bias of individual studies, including whether this will be done at the outcome or study level, or both; state how this information will be used in data synthesis	☒	☐	238-244
DATA					
**Synthesis**	15a	Describe criteria under which study data will be quantitatively synthesized	☒	☐	245-252
	15b	If data are appropriate for quantitative synthesis, describe planned summary measures, methods of handling data, and methods of combining data from studies, including any planned exploration of consistency (e.g., I^2, Kendall's tau)	☒	☐	245-252
	15c	Describe any proposed additional analyses (e.g., sensitivity or subgroup analyses, meta-regression)	☒	☐	245-252
	15d	If quantitative synthesis is not appropriate, describe the type of summary planned	☒	☐	245-252

For peer review only

BioMed Central The Open Access Publisher

Section/topic	#	Checklist item	Information reported Yes	Information reported No	Line number(s)
**Meta-bias(es)**	16	Specify any planned assessment of meta-bias(es) (e.g., publication bias across studies, selective reporting within studies)	☒	☐	245-252
**Confidence in cumulative evidence**	17	Describe how the strength of the body of evidence will be assessed (e.g., GRADE)	☒	☐	245-252

For peer review only

BioMed Central The Open Access Publisher

Appendix 2: Health Service Coverage Indicators

**Indicators for measuring progress towards reproductive, maternal, newborn and child health (RMNCH)**
**Family planning** (e.g., demand satisfied with modern contraception methods in women)
**Antenatal care** (e.g., women who received antenatal care four or more time)
**Delivery care** (e.g., births attended by skilled health personnel)
**Postnatal care** (e.g., women or newborn that received postnatal care services after birth)
**Child immunization** (e.g., children aged 1 year who have received three doses of diphtheria, tetanus, and pertussis vaccine (DPT3) vaccine)
**Health-seeking behaviour for pneumonia** (e.g., children with suspected pneumonia who have sought care)
**Indicators for measuring progress towards infectious diseases**
**Tuberculosis treatment** (e.g., people who received effective tuberculosis treatment)
**Human immunodeficiency virus (HIV) therapy** (e.g., people with HIV receiving antiretroviral treatment)
**Use of insecticide-treated bed nets for malaria prevention** (e.g., people sleeping under insecticide-treated nets)
**Adequate sanitation** (e.g., households using at least basic sanitation facilities)
**Neglected tropical diseases treatment and care (**e.g., people required treatment for any of the neglected tropical diseases). Neglected tropical diseases are a diverse group of 20 conditions including: Buruli ulcer, Chagas disease, dengue and chikungunya, dracunculiasis (Guinea-worm disease), echinococcosis, foodborne trematodiases, human African trypanosomiasis (sleeping sickness), leishmaniasis, leprosy (Hansen's disease), lymphatic filariasis, mycetoma, chromoblastomycosis and other deep mycoses, onchocerciasis (river blindness), rabies, scabies and other ectoparasitoses, schistosomiasis, soil-transmitted helminthiases, snakebite envenoming, taeniasis/cysticercosis, trachoma, and yaws and other endemic treponematoses.
**Indicators for measuring progress towards non-communicable diseases**
**Prevention and treatment of raised blood pressure** (e.g., prevalence of raised blood pressure regardless of treatment status)
**Prevention and treatment of raised blood glucose** (e.g., mean fasting plasma glucose)
**Cervical cancer screening** (e.g., prevalence of cervical cancer screening at aged 30-49)
**Tobacco (non-)smoking** (e.g., prevalence of tobacco use among adults aged at least 15 years)

For peer review only - http://bmjopen.bmj.com/site/about/guidelines.xhtml

Appendix 3. Electronic Search Strategies for Databases

MEDLINE via Ovid (searched on August 29, 2022; Period: 1946 - August 26, 2022)

Concept		Keyword	umber	Result
**Inequality**	**Controlled Vocabulary**	exp "gender identity"/ or exp "sexual and gender minorities"/ or exp "disabled persons"/ or exp geriatrics/ or "health equity"/ or "health inequities"/ or "healthcare disparities"/ or "health status disparities"/ or "health services accessibility"/ or "sex factors"/ or women/ or men/ or "residence characteristics"/ or "urban population"/ or "rural population"/ or "cultural characteristics"/ or "cultural diversity"/ or religion/ or "social discrimination"/ or "socioeconomic factors"/ or "vulnerable populations"/ or "social class"/ or "minority groups"/ or "ethnic and racial minorities"/ or "educational status"/ or unemployment/	1	884,458
	**Free Text**	(equit* or inequit* or inequalit* or disparit* or equalit* or deprivation or gini or "concentration index" or ((health* or medical) adj3 (availabl* or access*)) or (gender adj (difference? or identit* or role?)) or (sex adj (disparit* or difference? or role?)) or ((wom#n* or m#n*) adj role?) or "sexual minorit*" or "gender minorit*" or LGBT* or 2SLGBT* or urban or rural or ethnic* or religi* or ((social* or socio-economic or socioeconomic or economic or structural or material) adj3 (advantage* or disadvantage* or exclude* or exclusion or include* or inclusion or status or position or gradient* or hierarch* or class* or determinant*)) or (health adj3 (gap* or gradient* or hierarch*)) or SES or SEP or sociodemographic* or socio-demographic* or income or wealth* or poverty or "educational status" or "educational level?" or "level? of education" or "educational attainment" or ((well or better or higher or worse or less) adj (educated)) or unemploy* or "home owner*" or tenure or affluen* or "well off" or "better off" or "worse off").ti,ab,kw,kf	2	1,197,912
	**All**	1 or 2	3	1,739,958
**Health Service Coverage**	**Controlled Vocabulary**	"universal health care"/	4	243
	**Free Text**	(UHC or (universal adj2 (coverage or health* or care or access))).ti,ab,kw,kf	5	12,120
	**All**	4 or 5	6	12,186
**Africa**	**Controlled Vocabulary**	exp africa/ not ("guinea pig" or "guinea pigs" or "aspergillus niger").ti,ab,kw,kf,cp,in	7	308,111
	**Free Text**	((africa* or algeria or algerie or angola or benin or dahomey or botswana or bechuanaland or "burkina faso" or "burkina fasso" or "upper volta" or "haute volta" or burundi or urundi or cameroon or cameroun or "canary islands" or "cape verde" or cabo verde or "ubangi shari" or chad or tchad or comoros or "comoro islands" or comores or mayotte or congo or zaire or djibouti or "french somaliland" or egypt or "united arab republic" or eritrea or eswatini or swaziland or ethiopia or gabon or "gabonese republic" or gambia or ghana or "gold coast" or guinea or "ivory coast" or "cote d?ivoire" or "cote d' ivoire" or kenya or lesotho or basutoland or liberia or libya or libia or jamahiriya or jamahiryia or madagascar or "malagasy republic" or malawi or nyasaland or mali or mauritania or mauritius or morocco or ifni or mozambique or "portuguese east africa" or mocambique or namibia or niger or nigeria or principe or "sao tome" or reunion or rwanda or ruanda or senegal or seychelles or "sierra leone" or somalia or "south africa" or "st helena" or sudan or tanzania or tanganyika or togo or "togolese republic" or tunisia or tunisie or uganda or "western sahara" or zambia or zimbabwe or "indian ocean islands" or sahara or magreb or magrib) not ("guinea pig" or "guinea pigs" or "aspergillus niger")).ti,ab,kw,kf,cp,in	8	885,575
	**All**	7 or 8	9	921,268
**Total result**		**3 AND 6 AND 9**	**10**	**1,924**

For peer review only - http://bmjopen.bmj.com/site/about/guidelines.xhtml

Embase via Elsevier (searched on August 29, 2022)

Concept		Keyword	umber	Result
**Inequality**	**Controlled Vocabulary**	'gender identity'/exp or 'sexual and gender minority'/exp or 'disabled person'/exp or 'disabled person'/exp or 'health equity'/de or 'health disparity'/de or 'health care access'/de or 'health care availability'/de or 'sex difference'/de or 'female'/de or 'male'/de or 'residence characteristics'/de or 'urban population'/de or 'rural population'/de or 'cultural factor'/de or 'cultural diversity'/de or 'religion'/de or 'social discrimination'/de or 'socioeconomics'/de or 'vulnerable population'/de or 'disadvantaged population'/de or 'social class'/de or 'minority group'/de or 'ethnic group'/de or 'educational status'/de or 'unemployment'/de	#1	15,833,425
	**Free Text**	(equit* or inequit* or inequalit* or disparit* or equalit* or deprivation or gini or "concentration index" or ((health* or medical) NEAR/3 (availabl* or access*)) or (gender NEXT/1 (difference$ or identit* or role$)) or (sex NEXT/1 (disparit* or difference$ or role$)) or ((wom?n* or m?n*) NEXT/1 role$) or "sexual minorit*" or "gender minorit*" or LGBT* or 2SLGBT* or urban or rural or ethnic* or religi* or ((social* or socio-economic or socioeconomic or economic or structural or material) NEAR/3 (advantage* or disadvantage* or exclude* or exclusion or include* or inclusion or status or position or gradient* or hierarch* or class* or determinant*)) or (health NEAR/3 (gap* or gradient* or hierarch*)) or SES or SEP or sociodemographic* or socio-demographic* or income or wealth* or poverty or "educational status" or "educational level$" or "level$ of education" or "educational attainment" or ((well or better or higher or worse or less) NEXT/1 (educated)) or unemploy* or "home owner*" or tenure or affluen* or "well off" or "better off" or "worse off"):ti,ab,kw	#2	1,527,2697
	**All**	1 or 2	#3	16,237,327
**Health Service Coverage**	**Controlled Vocabulary**	'universal health care'/de	#4	600
	**Free Text**	(UHC or (universal NEAR/2 (coverage or health* or care or access))):ti,ab,kw	#5	14,728
	**All**	4 or 5	#6	14,913
**Africa**	**Controlled Vocabulary**	'africa'/exp not ("guinea pig" or "guinea pigs" or "aspergillus niger"):ti,ab,kw,ca,ad,ff	#7	397,519
	**Free Text**	((africa* or algeria or algerie or angola or benin or dahomey or botswana or bechuanaland or "burkina faso" or "burkina fasso" or "upper volta" or "haute volta" or burundi or urundi or cameroon or cameroun or "canary islands" or "cape verde" or cabo verde or "ubangi shari" or chad or tchad or comoros or "comoro islands" or comores or mayotte or congo or zaire or djibouti or "french somaliland" or egypt or "united arab republic" or eritrea or eswatini or swaziland or ethiopia or gabon or "gabonese republic" or gambia or ghana or "gold coast" or guinea or "ivory coast" or "cote d$ivoire" or "cote d? ivoire" or kenya or lesotho or basutoland or liberia or libya or libia or jamahiriya or jamahiryia or madagascar or "malagasy republic" or malawi or nyasaland or mali or mauritania or mauritius or morocco or ifni or mozambique or "portuguese east africa" or mocambique or namibia or niger or nigeria or principe or "sao tome" or reunion or rwanda or ruanda or senegal or seychelles or "sierra leone" or somalia or "south africa" or "st helena" or sudan or tanzania or tanganyika or togo or "togolese republic" or tunisia or tunisie or uganda or "western sahara" or zambia or zimbabwe or "indian ocean islands" or sahara or magreb or magrib) not ("guinea pig" or "guinea pigs" or "aspergillus niger")):ti,ab,kw,ca,ad,ff	#8	859,620
	**All**	7 or 8	#9	960,563
**Total result**		**#3 AND #6 AND #9**	**#10**	2,586

For peer review only - http://bmjopen.bmj.com/site/about/guidelines.xhtml

Web of Science (searched on August 29, 2022)

Concept		Keyword	umber	Result
**Inequality**	**Free Text**	TS=(equit* or inequit* or inequalit* or disparit* or equalit* or deprivation or gini or "concentration index" or ((health* or medical) NEAR/2 (availabl* or access*)) or (gender NEAR/0 (difference$ or identit* or role$)) or (sex NEAR/0 (disparit* or difference$ or role$)) or ((wom?n* or man* or men*) NEAR/0 role$) or "sexual minorit*" or "gender minorit*" or LGBT* or 2SLGBT* or urban or rural or ethnic* or religi* or ((social* or socio-economic or socioeconomic or economic or structural or material) NEAR/2 (advantage* or disadvantage* or exclude* or exclusion or include* or inclusion or status or position or gradient* or hierarch* or class* or determinant*)) or (health NEAR/2 (gap* or gradient* or hierarch*)) or SES or SEP or sociodemographic* or socio-demographic* or income or wealth* or poverty or "educational status" or "educational level$" or "level$ of education" or "educational attainment" or ((well or better or higher or worse or less) NEAR/0 (educated)) or unemploy* or "home owner*" or tenure or affluen* or "well off" or "better off" or "worse off")	#1	3,056,690
**Health Service Coverage**	**Free Text**	TS=(UHC or (universal NEAR/1 (coverage or health* or care or access)))	#2	15,460
**Africa**	**Free Text**	TS=((africa* or algeria or algerie or angola or benin or dahomey or botswana or bechuanaland or "burkina faso" or "burkina fasso" or "upper volta" or "haute volta" or burundi or urundi or cameroon or cameroun or "canary islands" or "cape verde" or cabo verde or "ubangi shari" or chad or tchad or comoros or "comoro islands" or comores or mayotte or congo or zaire or djibouti or "french somaliland" or egypt or "united arab republic" or eritrea or eswatini or swaziland or ethiopia or gabon or "gabonese republic" or gambia or ghana or "gold coast" or guinea or "ivory coast" or "cote d$ivoire" or "cote d' ivoire" or kenya or lesotho or basutoland or liberia or libya or libia or jamahiriya or jamahiryia or madagascar or "malagasy republic" or malawi or nyasaland or mali or mauritania or mauritius or morocco or ifni or mozambique or "portuguese east africa" or mocambique or namibia or niger or nigeria or principe or "sao tome" or reunion or rwanda or ruanda or senegal or seychelles or "sierra leone" or somalia or "south africa" or "st helena" or sudan or tanzania or tanganyika or togo or "togolese republic" or tunisia or tunisie or uganda or "western sahara" or zambia or zimbabwe or "indian ocean islands" or sahara or magreb or magrib) not ("guinea pig" or "guinea pigs" or "aspergillus niger")) or CU=((africa* or algeria or algerie or angola or benin or dahomey or botswana or bechuanaland or "burkina faso" or "burkina fasso" or "upper volta" or "haute volta" or burundi or urundi or cameroon or cameroun or "canary islands" or "cape verde" or cabo verde or "ubangi shari" or chad or tchad or comoros or "comoro islands" or comores or mayotte or congo or zaire or djibouti or "french somaliland" or egypt or "united arab republic" or eritrea or eswatini or swaziland or ethiopia or gabon or "gabonese republic" or gambia or ghana or "gold coast" or guinea or "ivory coast" or "cote d$ivoire" or "cote d' ivoire" or kenya or lesotho or basutoland or liberia or libya or libia or jamahiriya or jamahiryia or madagascar or "malagasy republic" or malawi or nyasaland or mali or mauritania or mauritius or morocco or ifni or mozambique or "portuguese east africa" or mocambique or namibia or niger or nigeria or principe or "sao tome" or reunion or rwanda or ruanda or senegal or seychelles or "sierra leone" or somalia or "south africa" or "st helena" or sudan or tanzania or tanganyika or togo or "togolese republic" or tunisia or tunisie or uganda or "western sahara" or zambia or zimbabwe or "indian ocean islands" or sahara or magreb or magrib) not ("guinea pig" or "guinea pigs" or "aspergillus niger")) or OG=((africa* or algeria or algerie or angola or benin or dahomey or botswana or bechuanaland or "burkina faso" or "burkina fasso" or "upper volta" or "haute volta" or burundi or urundi or cameroon or cameroun or "canary islands" or "cape verde" or cabo verde or "ubangi shari" or chad or tchad or comoros or "comoro islands" or comores or mayotte or congo or zaire or djibouti or "french somaliland" or egypt or "united arab republic" or eritrea or eswatini or swaziland or ethiopia or gabon or "gabonese republic" or gambia or ghana or "gold coast" or guinea or "ivory coast" or "cote d$ivoire" or "cote d' ivoire" or kenya or lesotho or basutoland or liberia or libya or libia or jamahiriya or jamahiryia or madagascar or "malagasy republic" or malawi	#3	2,204,863

For peer review only - http://bmjopen.bmj.com/site/about/guidelines.xhtml

		or nyasaland or mali or mauritania or mauritius or morocco or ifni or mozambique or "portuguese east africa" or mocambique or namibia or niger or nigeria or principe or "sao tome" or reunion or rwanda or ruanda or senegal or seychelles or "sierra leone" or somalia or "south africa" or "st helena" or sudan or tanzania or tanganyika or togo or "togolese republic" or tunisia or tunisie or uganda or "western sahara" or zambia or zimbabwe or "indian ocean islands" or sahara or magreb or magrib) not ("guinea pig" or "guinea pigs" or "aspergillus niger"))		
**Total result**		**#1 AND #2 AND #3**	**#4**	**2,145**

For peer review only

CINAHL via EBSCOhost (searched on August 19, 2022)

Concept		Keyword	umber	Result
**Inequality**	**Controlled Vocabulary**	(MH "gender identity+") or (MH "sexual and gender minorities+") or (MH "persons with disabilities+") or (MH "geriatrics+") or (MH "health inequities") or (MH "healthcare disparities") or (MH "health status disparities") or (MH "health services accessibility") or (MH "sex factors") or (MH "women") or (MH "men") or (MH "residence characteristics") or (MH "urban population") or (MH "rural population") or (MH "cultural diversity") or (MH "religion and religions") or (MH "discrimination") or (MH "socioeconomic factors") or (MH "special populations") or (MH "social class") or (MH "minority groups") or (MH "ethnic groups") or (MH "educational status") or (MH "unemployment")	S1	544,616
	**Free Text**	TI (equit* or inequit* or inequalit* or disparit* or equalit* or deprivation or gini or "concentration index" or ((health* or medical) N2 (availabl* or access*)) or (gender W1 (difference# or identit* or role#)) or (sex W1 (disparit* or difference# or role#)) or ((wom?n* or m?n*) W1 role#) or "sexual minorit*" or "gender minorit*" or LGBT* or 2SLGBT* or urban or rural or ethnic* or religi* or ((social* or socio-economic or socioeconomic or economic or structural or material) N2 (advantage* or disadvantage* or exclude* or exclusion or include* or inclusion or status or position or gradient* or hierarch* or class* or determinant*)) or (health N2 (gap* or gradient* or hierarch*)) or SES or SEP or sociodemographic* or socio-demographic* or income or wealth* or poverty or "educational status" or "educational level#" or "level# of education" or "educational attainment" or ((well or better or higher or worse or less) W1 (educated)) or unemploy* or "home owner*" or tenure or affluen* or "well off" or "better off" or "worse off") OR<br>AB (equit* or inequit* or inequalit* or disparit* or equalit* or deprivation or gini or "concentration index" or ((health* or medical) N2 (availabl* or access*)) or (gender W1 (difference# or identit* or role#)) or (sex W1 (disparit* or difference# or role#)) or ((wom?n* or m?n*) W1 role#) or "sexual minorit*" or "gender minorit*" or LGBT* or 2SLGBT* or urban or rural or ethnic* or religi* or ((social* or socio-economic or socioeconomic or economic or structural or material) N2 (advantage* or disadvantage* or exclude* or exclusion or include* or inclusion or status or position or gradient* or hierarch* or class* or determinant*)) or (health N2 (gap* or gradient* or hierarch*)) or SES or SEP or sociodemographic* or socio-demographic* or income or wealth* or poverty or "educational status" or "educational level#" or "level# of education" or "educational attainment" or ((well or better or higher or worse or less) W1 (educated)) or unemploy* or "home owner*" or tenure or affluen* or "well off" or "better off" or "worse off") OR<br>SU (equit* or inequit* or inequalit* or disparit* or equalit* or deprivation or gini or "concentration index" or ((health* or medical) N2 (availabl* or access*)) or (gender W1 (difference# or identit* or role#)) or (sex W1 (disparit* or difference# or role#)) or ((wom?n* or m?n*) W1 role#) or "sexual minorit*" or "gender minorit*" or LGBT* or 2SLGBT* or urban or rural or ethnic* or religi* or ((social* or socio-economic or socioeconomic or economic or structural or material) N2 (advantage* or disadvantage* or exclude* or exclusion or include* or inclusion or status or position or gradient* or hierarch* or class* or determinant*)) or (health N2 (gap* or gradient* or hierarch*)) or SES or SEP or sociodemographic* or socio-demographic* or income or wealth* or poverty or "educational status" or "educational level#" or "level# of education" or "educational attainment" or ((well or better or higher or worse or less) W1 (educated)) or unemploy* or "home owner*" or tenure or affluen* or "well off" or "better off" or "worse off")	S2	654,635
	**All**	S1 or S2	S3	874,600
**Health Service Coverage**	**Controlled Vocabulary**	MH "universal health care"	S4	552
	**Free Text**	TI (UHC or (universal N1 (coverage or health* or care or access))) OR AB (UHC or (universal N1 (coverage or health* or care or access))) OR SU (UHC or (universal N1 (coverage or health* or care or access)))	S5	6,489
	**All**	S4 or S5	S6	6,489

For peer review only - http://bmjopen.bmj.com/site/about/guidelines.xhtml

**Africa**	**Controlled Vocabulary**	(MH "africa+") not (TI ("guinea pig" or "guinea pigs" or "aspergillus niger") OR AB ("guinea pig" or "guinea pigs" or "aspergillus niger") OR SU ("guinea pig" or "guinea pigs" or "aspergillus niger") OR AF ("guinea pig" or "guinea pigs" or "aspergillus niger"))	S7	96,591
	**Free Text**	TI ((africa* or algeria or algerie or angola or benin or dahomey or botswana or bechuanaland or "burkina faso" or "burkina fasso" or "upper volta" or "haute volta" or burundi or urundi or cameroon or cameroun or "canary islands" or "cape verde" or cabo verde or "ubangi shari" or chad or tchad or comoros or "comoro islands" or comores or mayotte or congo or zaire or djibouti or "french somaliland" or egypt or "united arab republic" or eritrea or eswatini or swaziland or ethiopia or gabon or "gabonese republic" or gambia or ghana or "gold coast" or guinea or "ivory coast" or "cote d#ivoire" or "cote d' ivoire" or kenya or lesotho or basutoland or liberia or libya or libia or jamahiriya or jamahiryia or madagascar or "malagasy republic" or malawi or nyasaland or mali or mauritania or mauritius or morocco or ifni or mozambique or "portuguese east africa" or mocambique or namibia or niger or nigeria or principe or "sao tome" or reunion or rwanda or ruanda or senegal or seychelles or "sierra leone" or somalia or "south africa" or "st helena" or sudan or tanzania or tanganyika or togo or "togolese republic" or tunisia or tunisie or uganda or "western sahara" or zambia or zimbabwe or "indian ocean islands" or sahara or magreb or magrib) not ("guinea pig" or "guinea pigs" or "aspergillus niger")) OR<br>AB ((africa* or algeria or algerie or angola or benin or dahomey or botswana or bechuanaland or "burkina faso" or "burkina fasso" or "upper volta" or "haute volta" or burundi or urundi or cameroon or cameroun or "canary islands" or "cape verde" or cabo verde or "ubangi shari" or chad or tchad or comoros or "comoro islands" or comores or mayotte or congo or zaire or djibouti or "french somaliland" or egypt or "united arab republic" or eritrea or eswatini or swaziland or ethiopia or gabon or "gabonese republic" or gambia or ghana or "gold coast" or guinea or "ivory coast" or "cote d#ivoire" or "cote d' ivoire" or kenya or lesotho or basutoland or liberia or libya or libia or jamahiriya or jamahiryia or madagascar or "malagasy republic" or malawi or nyasaland or mali or mauritania or mauritius or morocco or ifni or mozambique or "portuguese east africa" or mocambique or namibia or niger or nigeria or principe or "sao tome" or reunion or rwanda or ruanda or senegal or seychelles or "sierra leone" or somalia or "south africa" or "st helena" or sudan or tanzania or tanganyika or togo or "togolese republic" or tunisia or tunisie or uganda or "western sahara" or zambia or zimbabwe or "indian ocean islands" or sahara or magreb or magrib) not ("guinea pig" or "guinea pigs" or "aspergillus niger")) OR<br>SU ((africa* or algeria or algerie or angola or benin or dahomey or botswana or bechuanaland or "burkina faso" or "burkina fasso" or "upper volta" or "haute volta" or burundi or urundi or cameroon or cameroun or "canary islands" or "cape verde" or cabo verde or "ubangi shari" or chad or tchad or comoros or "comoro islands" or comores or mayotte or congo or zaire or djibouti or "french somaliland" or egypt or "united arab republic" or eritrea or eswatini or swaziland or ethiopia or gabon or "gabonese republic" or gambia or ghana or "gold coast" or guinea or "ivory coast" or "cote d#ivoire" or "cote d' ivoire" or kenya or lesotho or basutoland or liberia or libya or libia or jamahiriya or jamahiryia or madagascar or "malagasy republic" or malawi or nyasaland or mali or mauritania or mauritius or morocco or ifni or mozambique or "portuguese east africa" or mocambique or namibia or niger or nigeria or principe or "sao tome" or reunion or rwanda or ruanda or senegal or seychelles or "sierra leone" or somalia or "south africa" or "st helena" or sudan or tanzania or tanganyika or togo or "togolese republic" or tunisia or tunisie or uganda or "western sahara" or zambia or zimbabwe or "indian ocean islands" or sahara or magreb or magrib) not ("guinea pig" or "guinea pigs" or "aspergillus niger")) OR<br>AF ((africa* or algeria or algerie or angola or benin or dahomey or botswana or bechuanaland or "burkina faso" or "burkina fasso" or "upper volta" or "haute volta" or burundi or urundi or cameroon or cameroun or "canary islands" or "cape verde" or cabo verde or "ubangi shari" or chad or tchad or comoros or "comoro islands" or comores or mayotte or congo or zaire or djibouti or "french somaliland" or egypt or "united arab republic" or eritrea or eswatini or swaziland or ethiopia or gabon or "gabonese republic" or gambia or ghana or "gold coast" or guinea or "ivory coast" or "cote d#ivoire" or "cote d' ivoire" or kenya or lesotho or basutoland or liberia or libya or libia or jamahiriya or jamahiryia or madagascar or "malagasy republic" or malawi or nyasaland or mali or mauritania or mauritius or morocco or ifni or mozambique or "portuguese east africa" or mocambique or	S8	210,457

For peer review only - http://bmjopen.bmj.com/site/about/guidelines.xhtml

		namibia or niger or nigeria or principe or "sao tome" or reunion or rwanda or ruanda or senegal or seychelles or "sierra leone" or somalia or "south africa" or "st helena" or sudan or tanzania or tanganyika or togo or "togolese republic" or tunisia or tunisie or uganda or "western sahara" or zambia or zimbabwe or "indian ocean islands" or sahara or magreb or magrib) not ("guinea pig" or "guinea pigs" or "aspergillus niger"))		
	**All**	S7 or S8	S9	210,575
**Total result**		**S3 AND S6 AND S9**	**S10**	**899**

For peer review only

For peer review only - http://bmjopen.bmj.com/site/about/guidelines.xhtml

PsycINFO via Ovid (searched on August 29, 2022; Period: 1806 - August Week 3, 2022)

Concept		Keyword	umber	Result
**Inequality**	**Controlled Vocabulary**	exp "gender identity"/ or exp "sexual minority groups"/ or exp disabilities/ or exp geriatrics/ or equity/ or "healthcare disparities"/ or "health care access"/ or "human sex differences"/ or "human females"/ or "human males"/ or neighborhoods/ or ghettoes/ or poverty areas/ or "rural environments"/ or "urban environments"/ or "minority groups"/ or "racial and ethnic differences"/ or "sociocultural factors"/ or "cross cultural differences"/ or "cultural identity"/ or "ethnic identity"/ or religion/ or "religious groups"/ or "social discrimination"/ or "socioeconomic factors"/ or "socioeconomic status"/ or "economic inequality"/ or "susceptibility (disorders)"/ or "at risk populations"/ or "social class"/ or disadvantaged/ or "educational attainment level"/ or unemployment/	1	619,023
	**Free Text**	(equit* or inequit* or inequalit* or disparit* or equalit* or deprivation or gini or "concentration index" or ((health* or medical) adj3 (availabl* or access*)) or (gender adj (difference? or identit* or role?)) or (sex adj (disparit* or difference? or role?)) or ((wom#n* or m#n*) adj role?) or "sexual minorit*" or "gender minorit*" or LGBT* or 2SLGBT* or urban or rural or ethnic* or religi* or ((social* or socio-economic or socioeconomic or economic or structural or material) adj3 (advantage* or disadvantage* or exclude* or exclusion or include* or inclusion or status or position or gradient* or hierarch* or class* or determinant*)) or (health adj3 (gap* or gradient* or hierarch*)) or SES or SEP or sociodemographic* or socio-demographic* or income or wealth* or poverty or "educational status" or "educational level?" or "level? of education" or "educational attainment" or ((well or better or higher or worse or less) adj (educated)) or unemploy* or "home owner*" or tenure or affluen* or "well off" or "better off" or "worse off").ti,ab,id.	2	698,649
	**All**	1 or 2	3	1,036,490
**Health Service Coverage**	**Free Text**	(UHC or (universal adj2 (coverage or health* or care or access))).ti,ab,id.	4	1,840
**Africa**	**Free Text**	((africa* or algeria or algerie or angola or benin or dahomey or botswana or bechuanaland or "burkina faso" or "burkina fasso" or "upper volta" or "haute volta" or burundi or urundi or cameroon or cameroun or "canary islands" or "cape verde" or cabo verde or "ubangi shari" or chad or tchad or comoros or "comoro islands" or comores or mayotte or congo or zaire or djibouti or "french somaliland" or egypt or "united arab republic" or eritrea or eswatini or swaziland or ethiopia or gabon or "gabonese republic" or gambia or ghana or "gold coast" or guinea or "ivory coast" or "cote d?ivoire" or "cote d' ivoire" or kenya or lesotho or basutoland or liberia or libya or libia or jamahiriya or jamahiryia or madagascar or "malagasy republic" or malawi or nyasaland or mali or mauritania or mauritius or morocco or ifni or mozambique or "portuguese east africa" or mocambique or namibia or niger or nigeria or principe or "sao tome" or reunion or rwanda or ruanda or senegal or seychelles or "sierra leone" or somalia or "south africa" or "st helena" or sudan or tanzania or tanganyika or togo or "togolese republic" or tunisia or tunisie or uganda or "western sahara" or zambia or zimbabwe or "indian ocean islands" or sahara or magreb or magrib) not ("guinea pig" or "guinea pigs" or "aspergillus niger")).ti,ab,id,in	5	141,449
**Total result**		**3 AND 4 AND 5**	**6**	**168**

For peer review only - http://bmjopen.bmj.com/site/about/guidelines.xhtml

Cochrane Library (searched on August 29, 2022)

Concept		Keyword	umber	Result
**Inequality**	**Controlled Vocabulary**	[mh "gender identity"] or [mh "sexual and gender minorities"] or [mh "disabled persons"] or [mh "geriatrics"] or [mh ^"health equity"] or [mh ^"health inequities"] or [mh ^"healthcare disparities"] or [mh ^"health status disparities"] or [mh ^"health services accessibility"] or [mh ^"sex factors"] or [mh ^"women"] or [mh ^"men"] or [mh ^"residence characteristics"] or [mh ^"urban population"] or [mh ^"rural population"] or [mh ^"cultural characteristics"] or [mh ^"cultural diversity"] or [mh ^"religion"] or [mh ^"social discrimination"] or [mh ^"socioeconomic factors"] or [mh ^"vulnerable populations"] or [mh ^"social class"] or [mh ^"minority groups"] or [mh ^"ethnic and racial minorities"] or [mh ^"educational status"] or [mh ^"unemployment"]	#1	18,168
	**Free Text**	(equit* or inequit* or inequalit* or disparit* or equalit* or deprivation or gini or "concentration index" or ((health* or medical) NEAR/2 (availabl* or access*)) or (gender NEXT (difference? or identit* or role?)) or (sex NEXT (disparit* or difference? or role?)) or ((wom?n* or m?n*) NEXT role?) or "sexual minorit*" or "gender minorit*" or LGBT* or 2SLGBT* or urban or rural or ethnic* or religi* or ((social* or socio-economic or socioeconomic or economic or structural or material) NEAR/2 (advantage* or disadvantage* or exclude* or exclusion or include* or inclusion or status or position or gradient* or hierarch* or class* or determinant*)) or (health NEAR/2 (gap* or gradient* or hierarch*)) or SES or SEP or sociodemographic* or socio-demographic* or income or wealth* or poverty or "educational status" or "educational level?" or "level? of education" or "educational attainment" or ((well or better or higher or worse or less) NEXT (educated)) or unemploy* or "home owner*" or tenure or affluen* or "well off" or "better off" or "worse off"):ti,ab,kw	#2	79,541
	**All**	#1 or #2	#3	87,932
**Health Service Coverage**	**Controlled Vocabulary**	[mh ^"universal health care"]	#4	0
	**Free Text**	(UHC or (universal NEAR/1 (coverage or health* or care or access))):ti,ab,kw	#5	306
	**All**	#4 or #5	#6	306
**Africa**	**Controlled Vocabulary**	[mh "africa"] not ("guinea pig" or "guinea pigs" or "aspergillus niger"):ti,ab,kw	#7	8,273
	**Free Text**	((africa* or algeria or algerie or angola or benin or dahomey or botswana or bechuanaland or "burkina faso" or "burkina fasso" or "upper volta" or "haute volta" or burundi or urundi or cameroon or cameroun or "canary islands" or "cape verde" or cabo verde or "ubangi shari" or chad or tchad or comoros or "comoro islands" or comores or mayotte or congo or zaire or djibouti or "french somaliland" or egypt or "united arab republic" or eritrea or eswatini or swaziland or ethiopia or gabon or "gabonese republic" or gambia or ghana or "gold coast" or guinea or "ivory coast" or "cote d?ivoire" or "cote d' ivoire" or kenya or lesotho or basutoland or liberia or libya or libia or jamahiriya or jamahiryia or madagascar or "malagasy republic" or malawi or nyasaland or mali or mauritania or mauritius or morocco or ifni or mozambique or "portuguese east africa" or mocambique or namibia or niger or nigeria or principe or "sao tome" or reunion or rwanda or ruanda or senegal or seychelles or "sierra leone" or somalia or "south africa" or "st helena" or sudan or tanzania or tanganyika or togo or "togolese republic" or tunisia or tunisie or uganda or "western sahara" or zambia or zimbabwe or "indian ocean islands" or sahara or magreb or magrib) not ("guinea pig" or "guinea pigs" or "aspergillus niger")):ti,ab,kw	#8	34,555
	**All**	#7 or #8	#9	34,555
**Total result**		**#3 AND #6 AND #9**	**#10**	**56**
